# An umbrella review of patient- and carer-reported measures for assessing adult end-of-life care quality outcomes

**DOI:** 10.1016/j.eclinm.2025.103516

**Published:** 2025-09-20

**Authors:** Chetna Malhotra, Louisa Poco, Shimoni Shah, Rowan H. Harwood, Jotheeswaran Thiyagarajan, Moise Muzigaba

**Affiliations:** a Lien Centre for Palliative Care, Duke-NUS Medical School, Singapore; b Program in Health Services and Systems Research, Duke-NUS Medical School, Singapore; c School of Health Sciences, University of Nottingham, Nottingham, NG7 2HA, United Kingdom; d Ageing and Health Unit, Department of Maternal, Newborn, Child, Adolescent Health and Ageing, World Health Organization, Geneva, Switzerland; e Department of Maternal, Newborn, Child, and Adolescent Health and Ageing, World Health Organization, Geneva, Switzerland

**Keywords:** PROMs, Quality of care, Quality of dying, Suffering, Quality of life, Umbrella review

## Abstract

**Background:**

Assessing end-of-life care quality is important yet selecting measures remains challenging. This umbrella review synthesizes evidence on measures for EOL outcomes—quality of life (QOL), care experience, quality of dying (QOD), and suffering.

**Methods:**

We searched five databases for systematic reviews published, 01 January 2009–29 July 2025. Included reviews assessed QOL, care experience, QOD and suffering measures for terminally ill adults and caregivers. We used AMSTAR2 to assess review quality and reported psychometrics per COSMIN. This study is registered on PROSPERO (CRD42024610359).

**Findings:**

From 30 reviews, we extracted 161 unique measures: 83 for QOL, 49 for care experience, 15 for QOD, and 14 for suffering. No single measure showed sufficient psychometric robustness. Among generic measures, those with stronger evidence included Palliative Care Outcome Scale (QOL); Family Assessment of Treatment at End-of-life, Care of the Dying Evaluation, and Quality of End-of-Life Care (care experience); Quality of Dying and Death Questionnaire (QOD); and Pictorial Representation of Illness and Self-Measure, and Patient Dignity Inventory (suffering).

**Interpretation:**

In this review, we recommended measures with stronger evidence and developed a checklist to guide selection based on domain relevance, disease group, population fit, and psychometrics. Future research should pursue rigorous validation and standardized reporting to strengthen assessment of end-of-life care.

**Funding:**

None.

**Research in context**

**Evidence before this study**

We searched PubMed from 01 January 2009 to 29 July 2025 for systematic reviews of measures assessing the quality of end-of-life care, using search terms such as “end-of-life”, “patient-reported outcome measures”, “quality of care”, “quality of dying”, “quality of life” and “suffering”. Only systematic reviews were included. Most identified reviews were narrowly focused, evaluating measures used in specific diseases (e.g., cancer, heart failure, renal disease, dementia), regions (e.g., China, Asia), care settings (e.g., hospital, hospice, intensive care), respondent types (e.g., patient-reported only), or singular constructs (e.g., quality of life or suffering). As a result, existing reviews offered limited generalizability and little consolidated guidance on selecting appropriate measures for use across diverse populations and settings. No prior review mapped available measures across the four core domains of end-of-life care quality: (1) quality of life, (2) care experience, (3) quality of dying, and (4) suffering.

**Added value of this study**

This is the first umbrella review to comprehensively identify and synthesize measures assessing the quality of end-of-life care across four key conceptual domains. We identified 161 unique measures from 30 systematic reviews. Based on what is reported in these systematic reviews, we highlight which measures have more consistent evidence of sufficient psychometric properties and map the domains they assess. We also critically appraise the methodological quality of the included reviews and identify key gaps in measure development, validation, and reporting.

**Implications of all the available evidence**

This umbrella review offers a comprehensive reference for researchers, clinicians, and policy makers selecting measures to assess end-of-life care quality. It supports informed measure selection, identifies current measurement gaps, and highlights priorities for future measure development and validation.

## Introduction

At the end of life (EOL), patients face risks of overtreatment, undertreatment or care that fails to align with their values and goals. In some regions, access to appropriate care is limited, while in others, economic incentives drive excessive interventions.^1^^,^^2^ All of these are indicators of poor-quality EOL care, affecting critical patient outcomes, including their quality of life (QOL), care experience, quality of dying (QOD) and suffering.

Improving EOL care starts with recognizing that *what cannot be measured cannot be improved*. Systematic measurement of EOL care quality is essential for providers to assess, compare, and evaluate interventions, with regular monitoring also being a goal for 2021–2030 UN Decade of Healthy Ageing.^3^ However, a key challenge remains: which scales or measures should be used to assess quality of EOL care? With a vast array of available measures, this is not an easy question to answer.

Recent systematic reviews have identified measures for quality of EOL care, but many focus on specific diseases^4^, ^5^, ^6^, ^7^, ^8^, ^9^, ^10^, ^11^, ^12^, ^13^ or geographical locations,^9^^,^^14^^,^^15^ settings,^9^^,^^14^, ^15^, ^16^, ^17^ target populations,^10^^,^^16^, ^17^, ^18^ or specific outcomes^19^ (e.g., care experiences) limiting the ability to select appropriate measures for different contexts. QOL measures, typically self-reported, assess how patients perceive and appraise their life situation across multiple dimensions.^20^ For patients unable to self-report, such as those with advanced dementia, proxy (caregiver or provider)-reported versions are available.^6^ Care experience measures, on the other hand, evaluate the perception, experiences or satisfaction of patients, caregivers or bereaved caregivers with elements of care received by patients.^21^ QOD measures, an extension of QOL measures, usually rely on proxy-reports and assess the patient's dying experience during the final days, weeks or month of life.^22^ Finally, suffering measures, which have gained prominence in recent years, focus on individual's state of severe distress related with events that threaten their personhood and integrity.^23^ A comprehensive synthesis of measures across these four outcomes would provide clarity on how to assess the quality of EOL care. These measures not only capture the outcomes and processes frequently assessed in EOL care research but also assess the domains consistently identified by patients and families as most important near EOL.^24^^,^^25^

We aimed to conduct an umbrella review of existing systematic reviews on measures for key outcomes of EOL care—QOL, care experience, QOD and suffering—providing a unified understanding of which measures to use for guiding improvements in care quality.

## Methods

### Search strategy and selection criteria

This umbrella review followed Cochrane's Preferred Reporting Items for Systematic Reviews and Meta-Analyses (PRISMA) guidelines. A systematic search in PubMed, CINAHL, EMBASE, Cochrane and Scopus covered systematic reviews published from 01 January 2009 to 29 July 2025. Reviews published before 01 January 2009 were excluded to minimize redundancy in tool extraction, as more recent reviews were likely to have already synthesized and incorporated findings from earlier literature. Eligible studies were systematic reviews assessing measures for adults (18+), with terminally or life limiting conditions, as defined by the review or by the Supportive and Palliative Care Indicators Tool,^26^ or their informal caregivers, focusing on QOL, suffering, QOD, and care experiences at EOL. Only English-language reviews were included; protocols, conference abstracts, theses, letters, editorials, non-systematic reviews were excluded. Reviews assessing only quality indicators—defined as system- or provider-level metrics that reflect structures, processes of care, or adherence to clinical standards (e.g., rates of hospice referral or documentation of advance care planning)—were excluded, as these differ from quality of EOL care measures, which directly assess patient- or caregiver-reported experiences.

The search used keywords–“end-of-life”, patient-reported outcome measures or “PROM”, “quality of care”, “quality of dying”, “quality of life” and “suffering” (details in Appendix). Two authors independently screened titles, abstracts and full text, resolving discrepancies with a third author. Rayyan was used to manage the screening process. Methodology is published in PROSPERO (CRD42024610359).

### Data extraction

Two authors independently extracted data for each unique measure, covering purpose, countries, language, target respondents, target population, setting, time frame of administration, domains, items, psychometric properties and methodological quality. Relevant measures were categorized by primary assessment purpose—QOL, care experience, quality of dying (QOD), or suffering. This distinction is not always absolute, as many measures span multiple domains. For example, some care experience measures, especially those assessed post-death, include items related to QOD (e.g., comfort at the time of dying), but primarily focus on experiences with health care providers. Similarly, many QOL measures encompass items that also reflect suffering. Our classification was therefore based on the primary purpose reported in the original validation study; when this was unclear, we applied our working conceptual framework. Another exception was the Palliative Care Outcome Scale (POS), which captures elements beyond traditional QOL domains, including needs assessment and care concerns. We classified it under QOL, in line with previous reviews, as it primarily measures patient outcomes rather than care experiences (or care delivery processes), suffering, or the final days of life (Table A1).

Target respondents included patients, informal caregivers, and health care providers (HCPs). Target populations were classified by specific illness or generic and setting as community-based or institutional. Domains were assessed based on the systematic review's classification. Measures not administered to patients, caregivers, or HCPs were excluded.

### Quality assessment of included reviews and evaluation of psychometric properties and methodological quality of measures

Two authors independently assessed quality of the included reviews using AMSTAR2,^27^ focusing on critical and non-critical domains (Table A2).

We extracted psychometric properties^28^ of the measures from the reviews when classified as sufficient or positive based on COSMIN. For interpretation of the threshold for “sufficient,” we present the original review criteria and a unified set of minimum threshold criteria for rating each property as sufficient, enabling comparison across reviews with differing rating systems (Table A3). To avoid misclassification, we do not report properties labelled as insufficient or indeterminate by the included reviews. Overall ratings for each property for each measure were determined using a majority rule.

We assessed the methodological quality of the studies included in reviews that assessed psychometric properties.^28^ Methodological qualities were classified using criteria in Table A4 allowing comparison across reviews using different rating standards. We summarized overall ratings for each property at the review level following the majority rule.

### Ethics

As this umbrella review was based on data extracted from previously published systematic reviews, ethical approval and informed consent were not required.

### Role of funding source

The authors received no financial support for the research, authorship, and/or publication of this article.

## Results

**Fig. 1:**
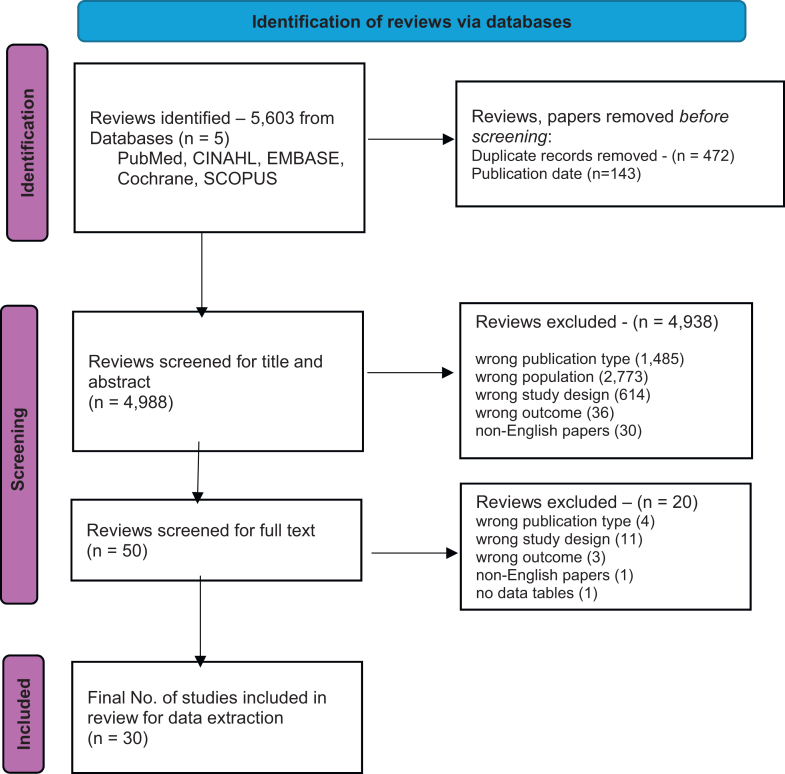
**PRISMA flow diagram**. Source: Page MJ, et al. BMJ 2021;372:n71. doi: 10.1136/bmj.n71.

Literature search results & selected review characteristics (Fig. 1, Table 1): We identified 5603 reviews from five databases, removing 472 duplicates and 143 reviews published before 01 January 2009, leaving 4998 reviews for title and abstract screening. From these, we selected 50 reviews for full-text screening, of which 30 were included. These reviews were published between March 2010^34^ and 19 June 2025,^25^ with search periods ranging from inception to December 2024.^19^ The number of unique measures included in these reviews ranged from 1^8^ to 35.^17^ Twelve reviews targeted specific populations–chronic kidney disease,^4^^,^^7^ dementia,^5^^,^^6^^,^^8^^,^^35^ cancer,^9^^,^^12^^,^^13^ end-stage liver disease,^11^ heart transplant,^10^ young adults,^25^ while others were generic. Three reviews focused on specific geographical setting—low-resource countries,^14^ Asia,^15^ China,^9^ and Portugal.^19^ A total of 161 unique measures, some with multiple versions, were extracted from the 30 reviews. Of these, 83 unique measures assessed QOL at EOL (Table 2), 49 evaluated care experience, 15 measured QOD, and 14 assessed suffering (Table 3).

**Table 1:** Systematic reviews included.

SR #	Author, year of publication	Search period	Type of measures included in the review	Number of unique relevant measures extracted
1.	Aiyegbusi et al. 2017^4^	Upto Dec 2015	Patient-reported measures (PROMs) for adults with chronic kidney disease	12
2.	Albers et al. 2010^29^	Jan 1990–April 2008	Quality of life in palliative care	15
3.	Bausewein et al. 2011^30^	upto Feb 2010	Use of Palliative care outcome scale and Support Team Assessment Schedule in clinical care and research	1
4.	Bowling et al. 2015^5^	upto July 2012	Quality of life for adults with dementia	16
5.	Burks et al. 2021^6^	Jan 1995–Sept 2020	Quality of life for adults with dementia	5
6.	Chambers et al. 2025^25^	Upto July 2023	Patient Reported Outcome Measures for young adults with life-limiting conditions	7
7.	Correia et al. 2011^31^	1999–2010	Quality of life in palliative care	10
8.	Ferreira et al. 2025^19^	Upto Dec 2024	Satisfaction with the healthcare received across different care settings	2
9.	Glover et al. 2011^7^	1950–2009	Quality of life for adults with end-stage kidney failure	9
10.	Gutierrez Sanchez et al. 2018^32^	1970–May 2016	Quality of dying	5
11.	Gutierrez-Sanchez et al. 2020^33^	Jan 1980–Sept 2019	Suffering in palliative care	6
12.	Hales et al. 2010^34^	1950–Nov 2008	Quality of dying	14
13.	Holden et al. 2015^35^	Jan 1965–Feb 2015	Patient-Reported Outcome Measures for Parkinson's Disease Dementia	3
14.	Hounsome et al. 2011^8^	1990–2009	EQ-5D in adults with dementia	1
15.	Kearns et al. 2017^36^	Jan 2006–July 2016	Quality of end-of-life care for adults with chronic disease	11
16.	Krikorian et al. 2013^37^	upto June 2011	Suffering in palliative care	8
17.	Kupeli et al. 2019^21^	upto May 2017	Quality of dying	20
18.	Lam et al. 2022^38^	upto Feb 2022	Dignity	3
19.	Lendon et al. 2015^18^	Jan 1990–June 2012	Patient Reported Experience Measures for family members or caregivers	11
20.	Li et al. 2023^9^	upto Aug 2022	Patient-Reported Outcome Measures for advanced cancer in China	14
21.	Li et al. 2024^39^	upto Jan 2023	Quality of life in life-limiting illnesses	11
22.	Mahmoudi et al. 2022^10^	Jan 2000–Dec 2020	Patient Reported Outcome Measures for heart transplant patients	11
23.	Peng et al., 2019^11^	Jan 1980–June 2018	Quality of life for end-stage liver disease	9
24.	Potts et al. 2018^14^	Oct 2016–Feb 2017	Palliative care outcome measures	3
25.	Quigley et al. 2020^16^	Jan 2000–March 2019	Patient Reported Experience Measures for patients and caregivers in hospice	5
26.	Roydhouse et al. 2017^12^	Not reported	Quality of life for advanced cancer	11
27.	Stiel et al. 2012^40^	upto Dec 2009	Palliative care outcome measures	20
28.	van Roij et al. 2018^13^	Jan 1990–Sept 2016	Quality of life for advanced cancer	19
29.	Virdun et al. 2023^17^	upto Sept 2022	Patient Reported Experience Measures for hospitalized patients with palliative care needs	35
30.	Xu et al. 2023^15^	upto April 2021	Quality of dying in Asia	3

**Table 2:** Measures assessing quality of life at the end of life.

#	Measure	# of studies	Respondents^b^	Population^c^	Setting^d^	# of items^e^	Domains included^f^	PP^a^
1.	Affect and Activity Ratings^5^	2	Patients, health care providers (HCPs), informal caregivers	Dementia	community, institution	21	activity domain: outside the home and indoors, affect domain: positive affect, negative affect	
2.	African Palliative Care Outcome Scale (African POS)^14^^,^^31^^,^^36^	10	patients	Generic	community, institution	10–14	physical, psychological, inter-personal and existential well-being	✔
3.	Alzheimer Disease-Related Quality of Life (ADRQL)^5^^,^^6^	18	HCPs, informal caregivers	Dementia	community, institution	24–48	social interaction, self-awareness: feelings and mood, enjoyment of activities, response to surroundings	
4.	Assessment of Quality of life at the EOL (AQOL)^13^	1	patients	Cancer		20	physical, psychological, social and spiritual well-being	✔
5.	Bath assessment of subjective quality of life in dementia (BASQID)^5^	4	patients	Dementia	community	14	self-rated overall QoL, health, memory, self-care, energy level, enthusiasm for doing things, social interactions, independence, usefulness	
6.	Brain Symptom and Impact Questionnaire (BASIQ)^13^	1	patients	Cancer		18	physical and psychological well-being	✔
7.	Brief Hospice Inventory (BHI)^29^^,^^36^	3	patients	Generic	institution	17	symptoms and QOL	✔
8.	Cambridge Palliative Audit Schedule (CAMPAS-R)^29^	1	patients	Generic	community	20	presence and interference of physical and psychological symptoms	✔
9.	Cancer Module Adolescent Form^25^	1	patients (YA), informal caregivers	Cancer (YA), blood disorders (YA)		27		✔
10.	Chinese Dialysis Quality of Life Scale (CDQOL)^4^	1	patients	Renal	community	29		
11.	CHOICE Health Experience Questionnaire (CHEQ)^4^	2	patients	Renal	community	83	physical functioning, body pain, general health, vitality, social functioning, emotional role, mental health. specific dimensions: freedom, travel restrictions, cognitive functioning, financial, restrictions on diet and fluids, recreation, work, body image, symptoms, sleep, sexual functioning, access-related problems, QOL	
12.	Chronic Heart Failure Questionnaire (CHFQ)^39^	2		Heart disease/failure		20	dyspnoea, fatigue, emotional status, mastery	
13.	Chronic Liver Disease Questionnaire^11^	14		Liver disease				
14.	Chronic Respiratory Questionnaire (CRQ)^39^	4		Respiratory disease	community	20	dyspnoea, fatigue, emotional status, mastery	
15.	City of Hope Quality of Life Survey^14^	1		Cancer		28		
16.	Client Generated Index (CGI)^34^	1	patients, informal caregivers	Cancer	community		comorbid conditions, up to five areas most impacted by illness	
17.	Cornell–Brown Scale for Quality of Life (CBS)^5^	3	patients, HCPs, informal caregivers	Dementia	community	19	negative affectivity (sadness, anxiety, irritability), physical complaints (weight loss, lack of energy), positive affectivity (serenity, self-esteem, happiness), satisfactions (weight satisfaction, restful sleep)	
18.	Darthmouth Cooperative functional health assessment chart and World Organization of General Practice/Family Physicians (COOP/WONCA)^12^	5	patients, informal caregivers	Cancer	community, institution	6	physical fitness, feelings, daily activities, social activities, overall health, pain, QOL	
19.	Dementia Quality of Life (DQOL)^5^	14	patients	Dementia	community, institution	30	self-esteem, positive affect/humour, negative affect, feelings of belonging, sense of aesthetics, overall QOL measure (optional)	
20.	Dementia Quality of Life (DEMQOL/DEMQOL-Proxy)^5^^,^^6^	11	patients, HCPs, informal caregivers	Dementia	community	28–31	daily activities, health and well-being, self-concept, cognitive functioning, social relationships	
21.	End-Stage Renal Disease Symptom Checklist (ESRD-SCL-TM)^7^	NR		Renal		43	limited physical and cognitive capacity, cardiac and renal dysfunction, corticosteroids side effects, increased growth of gum and hair, transplantation-related psychological distress	
22.	EORTC Quality of Life Questionnaire^9^^,^^12^^,^^13^^,^^29^^,^^31^^,^^40^	134	patients	Cancer	community, institution	15–36	role functioning, cognitive functioning, social functioning, fatigue, nausea/vomiting, pain, dyspnoea, insomnia, appetite loss, constipation, diarrhoea, financial difficulties	✔
23.	EuroQol 5 Dimension (EQ-5D)^4^^,^^7^^,^^8^^,^^10^, ^11^, ^12^, ^13^^,^^39^^,^^40^	49	patients, informal caregivers	Generic	community	5	mobility, self-care, usual activities, pain/discomfort, anxiety/depression, visual analog scale	✔
24.	Ferrans and Powers QOL Index (QLI 3.0)^7^^,^^25^	1	patients (YA)	Renal, Cancer (YA)		64		✔
25.	Functional Assessment of Chronic Illness Therapy-Palliative Care (FACIT-Pal)^13^^,^^29^^,^^31^^,^^39^	6	patients	Generic	community, institution	19–46	physical wellbeing, social/family well-being, emotional wellbeing, functional well-being, palliative care subscale	✔
26.	Functional Assessment of Cancer Therapy General (FACT-G, FACIT-Sp/FACT-C/FACT-Br/FAACT)^9^^,^^12^^,^^13^^,^^25^^,^^39^^,^^40^	39	patients, informal caregivers	Cancer (YA, adults)	community, institution	18–50	physical wellbeing, social/family well-being, emotional wellbeing, functional well-being	✔
27.	Gastrointestinal Quality of Life Index (GIQLI)^4^	1	patients	Kidney transplant	community	36	Gastrointestinal (GI) symptoms, emotional status, physical function, social function, strain of medical treatment	
28.	Generic Core Scales Adolescent Form^25^	1	patients (YA)	Cancer (YA), blood disorders (YA)		23		✔
29.	Health Related Quality of Life^40^	9		Generic				
30.	Heart Transplant Intervention Scale^10^	1	patients	Heart transplant		63	psychological state, social interaction, physical and occupational function, Information provision, self-care teaching, emotional/supportive, family-related, work/school/financial	
31.	Heart Transplant Stressor Scale (Jalowiec Stressor Scale)^10^	5	patients	Heart transplant		81	physical, psychological, self-care, family, work/school/financial, hospital/clinic	
32.	Hebrew Rehabilitation Centre for Aged Quality of Life (HRCA-QL)^31^^,^^40^	2	patients	Cancer		5	mobility, activities of daily life, health, support and prospects	
33.	Hepatitis Quality of Life Questionnaire (HQLQ)^11^	1		Hepatitis B/C				
34.	Hospice Quality of Life Index (HQLI)^12^^,^^13^^,^^29^^,^^40^	16	patients, informal caregivers	Generic	community, institution	25–28	social/spiritual; psychological/emotional: positive daily views, anger, loneliness, concern/worry for self, masculinity/femininity, pain relief, sadness, concern/worry for friends and family, sleep quality; physical/functional: constipation, engagement in enjoyable activities, ability to do usual work, tiredness, eating, financial	✔
35.	Hospice Quality of Life Scale (HQLS)^31^	1	patients	Cancer	community, institution	40	physical, psychological, and spiritual aspects, family and social economy and global aspects	
36.	Kansas City Cardiomyopathy Questionnaire (KCCQ/KCCQ-12)^10^^,^^39^	12	patients	Heart disease/failure	community, institution	‘12-23	physical limitation, symptom stability, symptom frequency, symptom burden, self-efficacy, QOL, social limitations	
37.	Kidney Disease Quality of Life (KDQOL/KDQOL-SF/KDQOL-36)^4^^,^^7^	32	patients	Renal	community	36–136	generic: physical functioning, physical role, bodily pain, general health, vitality, social functioning, emotional role, mental health; disease specific: symptoms/problems, effects of kidney disease on daily life, burden of kidney disease, work status, cognitive function, quality of social interaction, sexual function, sleep, social support, dialysis staff encouragement, patient satisfaction	
38.	Kidney Transplant Questionnaire (KTQ)^4^	4	patients	Kidney transplant	community, institution	25	physical symptoms–patient-specific such as fatigue, uncertainty/fear, appearance, emotional	
39.	Life Satisfaction Index (LSI)^7^	NR		Renal		13–20	zest for life, resolution and fortitude, congruence between desired and achieved goals, high physical psychological, and social self-concept, happy, optimistic mood tone	
40.	Linear Analog Scale Assessments (LASAs)^13^	1	patients	Cancer		5	physical, psychological and spiritual well-being	✔
41.	Liver Disease Quality of Life Questionnaire (LDQOL)^11^	2		Liver disease				
42.	Lung Cancer Symptom Scale (LCSS)^31^	1	patients	Cancer	community, institution	9	visual analogue scales–dimensions of physical and functionality (physical, cognitive and social aspects) and overall QOL (cognitive, psychological, social and spiritual)	
43.	McGill Quality of Life Questionnaire (MQOL/MQOL-HK/CSF)^9^^,^^12^^,^^13^^,^^29^^,^^31^^,^^40^	56	patients, informal caregivers	Cancer	community, institution	8–20	physical, psychological, existential wellbeing, and the domains of social and family support	✔
44.	McMaster Quality of Life Scale (MQLS)^29^^,^^40^	2	patients	Generic	community	32	physical, cognition, social, energy, role, rest, function, emotion	✔
45.	Medical Outcomes Study Short Form (SF-36/SF-36v2/SF-12/SF-6D)^4^^,^^7^^,^^10^, ^11^, ^12^^,^^25^^,^^39^^,^^40^	122	patients, informal caregivers	Generic, cancer (YA)	community	11–36	vitality, physical functioning, pain, general health, physical, emotional and social role functioning, mental health	✔
46.	Minnesota Living with Heart Failure Questionnaire (MLHFQ)^10^^,^^39^	9	patients	Heart disease/failure	institution	21	physical, emotional/psychologic functioning, socioeconomic	
47.	Missoula-VITAS Quality of Life Index (MVQOLI/MVQOLI-R)^13^^,^^29^^,^^31^^,^^36^^,^^40^	16	patients	Generic	community, institution	25–26	symptoms, functions, interpersonal, well-being, transcendent	✔
48.	Modified Chinese QoL questionnaire^11^	1		Liver disease				
49.	Modified City of Hope Patient Questionnaire (MCOHPQ)^36^	2	patients	Generic	institution	95	physical, emotional/relationship, spiritual, place of care/environment, care providers communication	✔
50.	Multiple Sclerosis Impact Scale (MSIS)^36^	4	patients	Multiple sclerosis	community, institution	29	QOL and impact of multiple sclerosis	✔
51.	National Institutes of Health Patient- Reported Outcomes Measurement Information System (NIH PROMIS)^13^	2	patients, informal caregivers	Cancer			physical, psychological, social, spiritual	✔
52.	Nottingham Health Profile (NHP)^4^^,^^7^^,^^11^	3	patients	Generic	community, institution	38	pain, energy, physical mobility, sleep, emotional reactions, social isolation	
53.	Observing QOL in Dementia (OQOLD) and Observing QOL for Dementia Advanced (OQOLDA)^5^	1	HCPs, informal caregivers	Dementia	community, institution	6		
54.	Organ Transplant Symptom and Well-being Instrument (OTSWI)^10^	2	patients	Heart transplant		8	fatigue, joint and muscle pain, cognitive functioning, basic activities of daily life, sleep problems, mood, foot pain, financial situation	
55.	Palliative Care Outcome Scale (POS)^13^^,^^29^, ^30^, ^31^^,^^36^	56	patients, HCPs, informal caregivers	Generic	community, institution	10–11	physical and psychological symptoms, spiritual considerations, practical and emotional concerns, psychosocial needs of patients and their families	✔
56.	Palliative Care Quality of Life Instrument (PQLI)^17^^,^^31^	2	patients	Cancer		28	dimensions of functionality, symptoms, choice of treatment (most important thing for the patient), psychological dimension and overall QOL.	
57.	Parkinson's Disease Quality of Life (PDQ-39/PDQ-8)^35^	1		Dementia				
58.	Patient Autonomy Questionnaire (PAQ)^29^	1	patients	Cancer		4–9	difficulty continuing usual activities and handing tasks over to others, dependency, loss of control over one's life, frustration as can do less than before, difficulties in continuing social activities, difficulties in asking for help, experiencing loss of control over one's own body, difficulties in making one's own decisions	✔
59.	Patient-Evaluated Problem Scores (PEPS)^13^	1	patients	Cancer			physical, psychological, social, spiritual well-being.	✔
60.	Progressive Deterioration Scale^5^	1	informal caregivers	Dementia		27	ability to travel distances alone and to leave immediate neighbourhood, confusion in familiar settings, use of familiar household implements, participation/enjoyment of leisure/cultural activities, extent to which does household chores, involvement in family finances/budgeting, interest in household tasks, taking public transport, self-care and routine tasks, social function/behaviour in social settings	
61.	Prostate Cancer Specific Quality of Life Instrument (PROSQOLI)^12^	2	patients, informal caregivers	Cancer			pain, physical activity, fatigue, appetite, constipation, family/romantic relationships, mood, passing urine, overall well-being, present pain intensity	
62.	Psychological Well-being in Cognitively Impaired Persons (PWBCIP)^5^	1	patients, informal caregivers	Dementia	community	11	positive interaction, frustrated/agitated, discontent	
63.	Quality of Life Instrument for Dementia (QUALIDEM)^5^^,^^6^	11	HCPs	Dementia	community, institution	18–37	care relationship, positive and negative affect, restless tense behaviour, positive self-image, social relations, social isolation, feeling at home, having something to do	
64.	Quality of Life and Health Questionnaire (QLHQ)^13^	1		Cancer		4	physical, psychological, social well-being	✔
65.	Quality of life assessment scale for gastric cancer patients (QLASTCM-Ga)^9^	1	patients	Cancer		43		✔
66.	Quality of Life Assessment Schedule (QOLAS)^5^	1	patients, informal caregivers	Dementia	community	10	physical, psychological, social/family, usual activities, cognitive	
67.	Quality of life at the EOL (QUAL-E/QUAL-E Fam)^13^^,^^17^^,^^29^^,^^39^^,^^40^	8	patients, informal caregivers	Generic		17–31	life completion, symptoms impact, relationship with health care provider, preparation for EOL	✔
68.	Quality of Life in Alzheimer's Disease (QOL-AD)^5^^,^^6^^,^^35^^,^^39^	62	patients, HCPs, informal caregivers	Dementia	community, institution	13–15	interpersonal relationships, financial difficulties, physical condition, memory, mood, overall health	
69.	Quality of Life in Late-Stage Dementia (QUALID)^5^^,^^6^	8	HCPs, informal caregivers	Dementia	community, institution	11	affective state, comfort, basic activities of life	
70.	Quality of Life in Life-Threatening Illness–Patient version (QOLLTI-P)^13^	1	patients	Cancer		1	physical, psychological, social, spiritual well-being	
71.	Quality of Life Index (QL/QLI 3.0I)^4^^,^^10^, ^11^, ^12^, ^13^	21	patients, informal caregivers	Generic	community, institution	5–68	health and functioning, psychological/spiritual status, social and economic aspects, family and relationships	✔
72.	Quality of Life Questionnaire for Dementia (QOL-D)^5^	2	patients, HCPs	Dementia	community, institution	31	positive and negative affect, actions, ability of communication, restlessness, attachment with others, spontaneity and activity	
73.	ReTransQoL (RTQ v1/RTQ v2)^4^	3	patients	Kidney transplant	institution	32–45	physical health, mental health, medical care, fear of losing graft, treatment	
74.	Schedule for the Evaluation of Individual Quality of Life (SEIQoL)^40^	6		Generic			important areas of life, level of functioning or satisfaction, relative importance to their overall QOL.	
75.	Self-reported 11-point QOL scale^39^	1		Generic		1	overall QOL score	
76.	Sickness Impact Profile (SIP)^7^^,^^10^^,^^11^	11		Generic		136	physical items: ambulation, mobility, and body care/movement; psychosocial items: social interaction, communication, alertness behaviour; emotional behaviour: home management, eating, sleep/rest, recreation and pastimes, and work	
77.	Spine Oncology Study Group Outcomes Questionnaire (SOSG-OQ)^13^	1	patients	Cancer		27	physical, psychological and social well-being	✔
78.	Spitzer Quality of Life Index (SQLI)^12^	3	patients, informal caregivers	Cancer	community		activity, daily living, health, support, outlook	
79.	St. Christopher's Index of Patient Priorities (SKIPP)^36^	8	patients	Generic	community, institution	8	QOL, concerns, change in concerns, impact of service on concerns	✔
80.	Structured Interview for Symptoms and Concerns in Palliative Care (SISC)^37^	2	patients	Cancer		13	physical, emotional, social, spiritual aspects and coping	✔
81.	Transplant Care Index (TCI)^10^	1	patients	Heart transplant		6	issues related to caring for a transplant, diet adherence, exercise, medication regimens	
82.	University of Washington Quality of Life Chinese Version (UWQOL-C)^9^	1	patients	Cancer		13	physical, social-emotional function	✔
83.	WHO Quality of Life (WHOQOL/WHOQOL-BREF)^4^^,^^7^^,^^10^^,^^12^^,^^14^^,^^40^	18	patients, informal caregivers	Generic	community	26–32	overall QOL, general and physical health, psychological, social relationships, environment	

Abbreviations: QOL, Quality of Life; QODD, Quality of dying and death; QOC, Quality of care; EOL, end of life; YA, young adults.

a PP, Psychometric properties.

b Respondents are categorized into: Patients (self-administered for PROMs), Informal caregivers (friends, any family member caring for patient, caregivers, surrogates), Healthcare professionals or HCPs (nurses, staff proxies, doctors, care teams).

c Population is categorized into disease groups: Dementia, Cancer, renal disease, heart disease or Generic. For measures with no specific disease group assigned, palliative care or mixed disease groups were clubbed as generic.

d Setting is categorized into Community (home-care setting, outpatient, memory or dialysis centers, geriatric facilities, day care, home-, outpatient- or community-based palliative care or community-based centers), Institution (Hospital-based setting such as ICU, inpatient, medical centers and nursing homes, assisted living and long-term care facilities, inpatient palliative care, hospice care). If specified as just palliative care it was categorized as both community/inpatient.

e Range of items based on reviews included.

f Domains included can be based on the scales of the instrument itself, or from the information extracted from the reviews included.

**Table 3:** Measures assessing care experience, quality of dying and suffering.

#	Measure	# of studies	Respondents^b^	Population^c^	Setting^d^	# of items^e^	Domains included^f^	PP^a^
***Care experience***								
1.	Bereaved Family Survey (BFS)^17^	1	informal caregivers	Generic	institution	17	respectful and compassionate care, communication, physical care, assessment and care planning	
2.	Canadian Health Care Evaluation Project (CANHELP)^17^	2	patients, informal caregivers	Generic		38–40	respectful and compassionate care, communication and teamwork, meaning and identity, physical care, assessment and care planning, symptom management, patient safety, supported access to clinicians	
3.	Care Evaluation Scale (CES/CES-10)^17^^,^^21^^,^^40^	6	bereaved informal caregivers	Cancer	community, institution	10–28	physical care by physician and nurse, psycho-existential care; help with decision-making for patient and for family, environment, family burden, cost, availability, coordination and consistency	✔
4.	Care of the Dying Evaluation (CODE)^17^^,^^21^	2	Bereaved informal caregivers	Generic	community	30–42	environment, communication, care	✔
5.	Caregiver Evaluation of the Quality of End-of-Life care (CEQUEL)^21^	1	informal caregivers	Cancer	community	13	prolonging death, perceived suffering, shared decision-making, preparation for death	✔
6.	Caregiver Voice Survey^17^	1	informal caregivers	Generic		62	respectful and compassionate care, communication, family involvement, maintaining role, meaning and identity, physical care, assessment and care planning, symptom management	
7.	Chinese Patient Satisfaction Questionnaire (ChPSQ-9)^9^	1	patients	Cancer		9	doctor and nurse related issues	
8.	Considerate^17^	1	patients	Generic		8	respectful and compassionate care, communication, assessment and care planning, symptom management, structural factors-patient	
9.	Consumer Assessment of Healthcare Providers and Systems (CAHPS) Cancer Care/Hospice Survey^17^	2	patients, informal caregivers	Cancer	community, institution	47–85	respectful and compassionate care, communication, family involvement, assessment and care planning, symptom management, teamwork	
10.	Consumer Quality Index Palliative Care (CQ-Index-PC)^17^^,^^21^	3	bereaved informal caregivers, patients	Generic	community, institution	22–33	patient and carer psychological spiritual wellbeing, attitudes to relatives, autonomy, provision of information, expertise	✔
11.	Dementia Care Mapping (DCM)^5^	5	HCPs	Dementia	community, institution		well/ill-being, social withdrawal activity	
12.	Dying Care Process Scale^17^	1	Bereaved informal caregivers	Generic		8	respectful and compassionate care, family environment, symptom management	
13.	End-of-life in Dementia Scales–SWC-EOLD^17^^,^^18^^,^^21^	13	informal caregivers	Dementia	institution	10	Decision-making, communication with HCPs, understanding condition, nursing care;	✔
14.	EORTC Satisfaction with In-Patient Cancer Care (EORTC IN-PATSAT32)^9^	1	patients	Cancer		32	HCP's technical skills, interpersonal skills, information provision and availability, other hospital staff members' interpersonal skills, wait times, hospital access, information exchange, hospital comfort, and overall satisfaction	✔
15.	euroQ2 Satisfaction with Care in the ICU^17^	1	informal caregivers	Generic	institution	33	respectful and compassionate care, communication, assessment and care planning, symptom management	
16.	Evaluating Care and Health Outcomes-for the Dying (ECHO-D)^21^	2	Bereaved informal caregivers	Cancer	institution	91–121	ward environment, care, facilities, communication	✔
17.	Family Assessment of Treatment of End-of-Life (FATE/FATE-S-14/FATE-S-12/FATE-32)^17^^,^^18^^,^^21^	10	informal caregivers	Generic	community, institution	12–58	well-being and dignity, information and communication, respect for treatment preferences, emotional and spiritual support, symptom management, inpatient facility, care around time of death	✔
18.	Family Evaluation of Hospice Care (FEHC)^17^^,^^21^	9	Bereaved informal caregivers	Generic	institution	56–61	information and care planning, provider care, symptom management, overall experience, spiritual, religious and existential, psychosocial care, caregiver support, responsiveness and timing, other, personal care, bereavement support, financial needs	
19.	Family Evaluation of Palliative Care (FEPC)^17^	1	informal caregivers	Generic	institution	45	respectful and compassionate care, communication, teamwork, family involvement, physical care, assessment and care planning, symptom management, supported access to clinicians	
20.	Family Perceptions of Care Scale (FPCS)^21^	3	informal caregivers	Generic	institution	25–27	resident care, family support, communication, rooming	✔
21.	Family Perceptions of Physician-Family Caregiver Communication (FPPFC)^21^	2	informal caregivers	Dementia	institution	7		✔
22.	Family Satisfaction in the ICU (FS-ICU)^16^, ^17^, ^18^^,^^40^	8	Bereaved informal caregivers	Generic	institution	25–34	total satisfaction, care satisfaction and decision-making satisfaction	
23.	Family Satisfaction with Advanced Cancer Care (FAMCARE/FAMCARE-5/FAMCARE-10/FAMCARE-patient/FAMCARE-2)^17^, ^18^, ^19^^,^^21^^,^^40^	25	patients, informal caregivers	Cancer	community, institution	5–30	Information giving, physical patient care, psychosocial care, availability of care	✔
24.	Family Satisfaction with Care Questionnaire^16^	1	informal caregivers	Generic	institution	35	emotional support, symptom management, HCP communication and competence, overall satisfaction, accessibility of information, involvement in decision making, satisfaction with death and dying	
25.	Feeling Heard and Understood^17^	1	patients	Cancer		2	communication	
26.	Health Competence Beliefs Inventory (HCBI)^25^	2	patients (YA), informal caregivers	Cancer (YA)		21	health perceptions, satisfaction with healthcare, cognitive competence, autonomy	✔
27.	Maastricht Instrument on Satisfaction with Terminal Care (MITTZ)^40^	1		Generic				
28.	McCusker EOLC scale^21^	1	informal caregivers	Generic		42	satisfaction, availability and continuity of care, physician qualities, availability and competence, communication with physician, preference for home care and physician decisions, involvement of patient-family in treatment decisions, freedom from pain, pain control	✔
29.	Otani et al., 2020 study developed questionnaire^17^	1	informal caregivers	Cancer		12	maintaining role, meaning and identity, patient safety, structural factors–patient	
30.	Patient and Family Satisfaction Survey^16^	1	patients, informal caregivers	Generic	institution	9	referral timeliness, pain and symptom control, communication, effort to assist patient in coping with EOL issues, respect for individualized care, discharge planning, respect for continuing or discontinuing treatments, spiritual concerns with EOL issues	
31.	Patient Satisfaction Questionnaire (PSQ)^17^	3	patients	Generic		12	respectful and compassionate care, effective communication	
32.	Postmortem Questionnaire-Short Form (QPM-SF)^21^	1	informal caregivers	Cancer	community, institution	37	integrated home care, hospice, physical care-information-global evaluation, needs	✔
33.	Quality Care Questionnaire-Palliative Care (QCQ-PC)^9^^,^^17^	2	patients	Cancer		32	communication with medical staff, discuss goals and plans of treatment and care, support and evaluation of overall care, accessibility and continuity of care	✔
34.	Quality from the Patient's Perspective (QPP-PC)^17^	1	patients	Generic	community, institution	51	respectful and compassionate care, communication, family involvement, maintaining role, meaning and identity, physical care, assessment and care planning, symptom management, supported access to clinicians, structural factors—patient	
35.	Quality Measure for Palliative Nursing^36^	1	patients	Generic	community	15	personal characteristics, communication skills, knowledge, relationship with patient and providing comfort	✔
36.	Quality of Communication Questionnaire (QOC)^17^^,^^36^	3	patients	Generic	institution	13–18	general communication skills, communication about EOL care	✔
37.	Quality of End-of-Life Care and Satisfaction with Treatment (QUEST)^17^^,^^18^^,^^40^	6	patients, informal caregivers	Generic	institution, community	15–47	Physician and satisfaction, nursing care and satisfaction	
38.	Quality of End-of-Life Care Questionnaire (QEOLC)^17^^,^^36^	4	patients, informal caregivers	Generic	institution	11–26	patient-centred systems, communication skills, symptom skills, affective skills, patient-centred values	✔
39.	Quality of oncology nursing care scale (QONCS)^9^	1	patients	Cancer		28	support and confirmation, spiritual care, belonging, value, respect	✔
40.	Reid-Gundlach Satisfaction with Services instrument^16^	2	patients	Generic	community	12	satisfaction of services, perception of service providers, likelihood of positive recommendations of services to others	
41.	Satisfaction Scale for Family members receiving Inpatient Palliative Care (Sat-Fam-IPC)^17^^,^^18^^,^^21^	4	Bereaved informal caregivers	Generic	community, institution	34–60	nursing care, facility, information, availability, family care, cost, symptom palliation	✔
42.	Satisfaction with care Scale^40^	1		Generic				
43.	Satisfaction with Doctors Questionnaire^17^	1	patients	Generic		8	respectful and compassionate care, communication	
44.	Sense of Security in Care—Patients' Evaluation (SEC-P)^36^	1	patients	Generic	community	15		✔
45.	The Sinclair Compassion Questionnaire (SCQ)^17^	1	patients	Generic		15	respectful and compassionate care, communication, assessment and care planning	
46.	Toolkit After-Death Bereaved Family Member Interview^17^^,^^18^^,^^21^	13	Bereaved informal caregivers	Generic	community, institution	36–74	physical comfort, emotional support, shared decision making, ACP, focus on individual, emotional and spiritual needs of family, care coordination, supporting self-efficacy of family, overall rating for patient focused, family centred care	✔
47.	Users' Satisfaction with Nursing Care instrument (SUCEH21)^19^	1	patients	Generic	community	NR	perception of healthcare quality, presence of a reference visitor, satisfaction with nursing care	
48.	Victorian Palliative Care Satisfaction Instrument (VPCSI)^17^	2	patients, informal caregivers	Generic	community, institution	58	respectful and compassionate care, communication, teamwork, family involvement, technical competence, symptom management, supported access to clinicians, structural factors-patient	
49.	Views of Informal Carers Evaluation of Services (VOICES)^17^^,^^18^	5	Bereaved informal caregivers	Generic	Institution	45–59	information and care planning, provider care, symptom management, overall experience, personal care, bereavement support	
***Quality of dying***								
1.	Cohen et al. 2005^34^	1	informal caregivers	Renal	community	2	peaceful death, died with dignity	
2.	Dialysis Discontinuation Quality of Dying (DDQOD)^32^^,^^34^	3	Health care providers (HCPs)	Renal	community		duration to death after decision to stop dialysis, presence of suffering (pre-existing and new), psychosocial domain (circumstances leading to the decision, level of consciousness, capacity to communicate, and presence of family/friends at the time of death)	
3.	Dying Care Outcomes Scale^17^	1	Bereaved informal caregivers	Generic		8	respectful and compassionate care, family environment, symptom management	
4.	End-of-life in Dementia Scales–CAD-EOLD^17^^,^^18^^,^^21^^,^^40^	13	informal caregivers, HCPs	Dementia	institution	14	physical distress, dying symptoms, emotional distress, well-being	✔
5.	McCanse Readiness for Death Instrument (MRDI)^29^	1		Generic	Institution	26	physiological, psychological, sociological, spiritual	✔
6.	Flacker et al. 2001^34^	1	HCPs	Generic	institution	1	quality of death	
7.	Ganzini et al. 2003^34^	1	HCPs	Generic	institution	1	quality of process of dying	
8.	Good Death Inventory (GDI/GDI-sv)^15^^,^^21^^,^^32^^,^^34^	12	HCPs, informal caregivers	Generic	institution	54	environmental, physical and psychological comfort, life completion, dying in a favourite place, maintaining hope and pleasure, independence, good relationship with medical staff and family, not being a burden to others, respect, religious and spiritual comfort, receiving enough treatment, control over the future, feeling that one's life is worth living, unawareness of death, pride and beauty, natural death, preparation for death	✔
9.	Good Death Scale (GDS)^15^^,^^32^^,^^34^	8	HCPs, informal caregivers	Cancer	community, institution	5	awareness that one is dying, acceptance of dying peacefully, honouring of patient's wishes, death timing, degree of physical comfort three days before death	✔
10.	Peruselli et al. 1999^34^	1	HCPs	Generic	community	12	desired place of death, presence of someone at the time of death, peacefulness, symptom control, the need for total pharmacological sedation in the last 12 h of life, state of consciousness, patient's awareness of the situation, performance of lifesaving procedures, rituals related to death, family members contacting the team after the patient's death, invasive procedures, instruments implemented at the time of death	
11.	Quality of Dying (QOD-Hospice/QOD-LTC)^21^^,^^32^^,^^34^	7	HCPs, informal caregivers	Generic	institution	11–23	completion, relationship with the healthcare system, preparation and security, symptom impact, social connection	✔
12.	QODD Questionnaire (QODD/QODD-ESP/QODD-D-ANG/QODD-D-MA/QODD-Chinese/QODD-Korean)^15^, ^16^, ^17^, ^18^^,^^21^^,^^29^^,^^32^^,^^34^^,^^40^	37	HCPs, informal caregivers	Generic	community, institution	12–48	symptoms and personal control, preparation for death, moment of death, family concerns, treatment preferences, whole person concerns	✔
13.	Ray et al. 2006^34^	1	HCPs, informal caregivers	Cancer	institution	1		
14.	Reynolds et al. 2002^34^	1	HCPs, informal caregivers	Generic	institution	1	whether or not they believed the resident's dying experience was a ‘good death’, how he/she would have wanted it	
15.	The Quality of Death^34^	2	informal caregivers	Cancer	community, institution	13	presence of significant others, physical ability, freedom from pain, peace/happiness, ability to maintain normal activities and to stay at home as long as wanted, to be at peace with God, to die in sleep, to be mentally alert, to complete tasks, to accept death, to know when death is imminent, to live until a key event has occurred	
***Suffering***								
1.	Cancer Distress Scales for Adolescents and Young Adults (CDS-AYA)^25^	3	patients (YA)	Cancer (YA)		49	impact of cancer: physical, impact of cancer: emotional, cognitive, cancer worry, mood	✔
2.	Initial Assessment of Suffering (IAS)^33^^,^^37^	2	patients	Cancer	institution	20–43	mood-factor, gastrointestinal symptoms, fears and family worries, knowledge and involvement, support-factor	✔
3.	Measurement Instrument for Dignity Amsterdam (MIDAM)^38^	1		Generic	community, institution	26	evaluation of self in relation to others, functional status, mental state, care and situational aspects	
4.	Mini-Suffering State Examination (MSSE)^33^^,^^37^	8	patients, HCPs, informal caregivers	Generic	institution	10	physical, psychological, social and spiritual factors	✔
5.	Palliative Patient's Dignity Scale (PPDS)^38^	1		Generic	community	8	dignity preservation, dignity threat	
6.	Patient Dignity Inventory (PDI)^13^^,^^29^^,^^38^	24	patients	Generic	institution	25	symptom and existential distress, dependency, peace of mind, social support	✔
7.	Peace, Equanimity, and Acceptance in the Cancer Experience (PEACE)^13^	1	patients	Cancer		12	psychological, spiritual well-being	✔
8.	Pictorial Representation of Illness and Self-Measure (PRISM/PRISM-/PRISM-R2)^33^^,^^37^	25	patients	Generic	institution	1	self-illness separation, illness perception	✔
9.	State Of Suffering-Five (SOS-V)^33^^,^^37^	2	patients	Cancer	community	69	signs and symptoms, loss of functions, personal aspects, social environment, nature and prognosis of disease	✔
10.	Suffering Assessment Questionnaire in Adults with Chronic Diseases or Life-Threatening Illness (SAQ)^33^	1	patients	Generic	community, institution	12	intrapersonal and interpersonal suffering, awareness of suffering, and spiritual suffering	✔
11.	Suffering Assessment Tool (SAT)^37^	1	patients	Generic		10	physical, spiritual, emotional/personal and familial	✔
12.	Suffering Scale^37^	1	patients	Pelvic pain		10		✔
13.	The Suffering Pictogram^33^	2	patients	Generic	institution	8	sensory (discomfort), emotional (worry, fear, anger, and sadness), cognitive (hopelessness and difficulty in acceptance), and spiritual (emptiness).	✔
14.	The Suffering Scales^37^	3	patients, informal caregivers	Generic		33	physical, psychological and existential	✔

a PP- Psychometric properties, EOL, end of life; YA, young adults.

b Respondents are categorized into: Patients (self-administered for PROMs), Informal caregivers (friends, any family member caring for patient, caregivers, surrogates), Healthcare professionals or HCPs (nurses, staff proxies, doctors, care teams).

c Population is categorized into disease groups: Dementia, Cancer, renal disease, heart disease or Generic. For measures with no specific disease group assigned, palliative care or mixed disease groups were clubbed as generic.

d Setting is categorized into Community (home-care setting, outpatient, memory or dialysis centers, geriatric facilities, day care or community-based centers), Institution (Hospital-based setting such as ICU, inpatient, medical centers and nursing homes, assisted living and long-term care facilities), and Hospice/Palliative care (Palliative care/hospice care setting, even if specified at home/inpatient or community setting).

e Range of items based on reviews included.

f Domains included can be based on the scales of the instrument itself, or from the information extracted from the reviews included.

Methodological quality of reviews (Table A2): We assessed 13 of the 16 AMSTAR2 domains (5 critical and 8 non-critical),^27^ as none of the reviews conducted a meta-analysis. Four reviews^4^^,^^15^^,^^33^^,^^39^ passed all critical domains, with three^4^^,^^33^^,^^39^ also passing all the non-critical domains. Ten reviews^4^^,^^6^^,^^13^^,^^15^^,^^19^^,^^21^^,^^32^^,^^33^^,^^38^^,^^39^ passed ten or more domains, while three^7^^,^^10^^,^^35^ failed all critical domains. All reviews were included for synthesis.

Psychometric properties and methodological qualities of measures (Tables 4 and 5): Nine reviews^9^^,^^13^^,^^15^^,^^21^^,^^25^^,^^29^^,^^33^^,^^36^^,^^37^ reported psychometric properties, with two^15^^,^^33^ using the 2018 COSMIN criteria.^41^ Sufficient evidence was found for internal consistency in 32 unique measures, reliability in 17, content validity in 46, hypothesis testing in 37, structural validity in six, responsiveness in five, and criterion validity, measurement error and cross-cultural validity in one.

**Table 4:** Psychometric properties rated sufficient in measures.

Tool #	Tool name	Disease group	Internal consistency	Reliability	Content validity	Structural validity	Hypothesis testing	Responsiveness	Cross cultural validity	Criterion validity	Measurement error
***Quality of Life***											
1.	African Palliative Outcome Scale (African POS)	Generic		+^36^	+^36^		+^36^				
2.	Assessment of Quality of life at the EOL (AQOL)	Cancer	+^13^		+^13^		+^13^				
3.	Brain Symptom and Impact Questionnaire (BASIQ)	Cancer			+^13^						
4.	Brief Hospice Inventory (BHI)	Generic	+^29^^,^^36^	+^36^							
5.	Cambridge Palliative Audit Schedule (CAMPAS-R)	Generic			+^29^						
6.	Cancer Module Adolescent Form^a^	Cancer, blood disorders	+^25^				+^25^				
7.	EORTC Quality of Life Questionnaire (QLQ) Core 30–EORTC QLQC30 (and a shortened version)	Cancer			+^13^		+^13^				
	EORTC QLQ-Spiritual Wellbeing 27 items–EORTC QLQ-SWB27	Cancer	+^9^		+^9^						
	EORTC QLQ Bone Metastases module–EORTC QLQBM22	Cancer		+^13^	+^13^		+^13^	+^13^			
	EORTC QLQ-STO22	Cancer	+^29^		+^29^						
	EORTC QLQ in patients with oesophageal cancer 18 items–EORTC QLQ-OES18	Cancer			+^29^						
	EORTC Core Questionnaire Oral Health–EORTC QLQOH17	Cancer			+^13^						
	EORTC QLQ-C15-PAL	Cancer			+^13^		+^13^				+^13^
8.	Functional Assessment of Chronic Illness Therapy-Palliative Care (FACIT-Pal)	Generic			+^13^		+^29^				
9.	Functional Assessment of Cancer Therapy—Colorectal (FACT-C)	Cancer					+^9^				
	Functional Assessment of Cancer Therapy General (FACT-G)	Cancer					+^13^				
	Functional assessment of Cancer Therapy-Brain (FACT-Br)	Cancer		+^13^	+^13^						
	Functional Assessment of Anorexia Cachexia Therapy (FAACT)	Cancer					+^13^				
10.	Generic Core Scales Adolescent Form^a^	Cancer, blood disorders	+^25^				+^25^				
11.	Hospice Quality of Life Index (HQLI)	Generic	+^29^				+^29^				
12.	Linear Analog Scale Assessments (LASAs)	Cancer			+^13^						
13.	McGill Quality of Life Questionnaire (MQOL)	Cancer	+^29^	+^29^	+^9^^,^^13^^,^^29^		+^9^^,^^29^				
	McGill Quality of Life Questionnaire-Cardiff Short Form (MQOL-CSF)	Cancer			+^13^		+^13^^,^^29^				
14.	McMaster Quality of Life Scale (MQLS)	Generic			+^29^		+^29^				
15.	Missoula-VITAS Quality of Life Index (MVQOLI, MVQOLI-R)	Generic			+^13^^,^^29^^,^^36^		+^29^	+^13^			
16.	Multiple Sclerosis Impact Scale (MSIS)	Multiple Sclerosis	+^36^	+^36^	+^36^		+^36^				
17.	Palliative Care Outcome Scale (POS)	Generic	+^36^	+^36^	+^13^^,^^36^		+^13^^,^^36^				
18.	Patient Autonomy Questionnaire (PAQ)	Cancer					+^29^				
19.	Quality of Life and Health Questionnaire (QLHQ)	Cancer					+^13^				
20.	Quality of life assessment scale for gastric cancer patients (QLASTCM-Ga)	Cancer	+^9^	+^9^	+^9^		+^9^				
21.	Quality of life at the EOL (QUAL-E)	Generic	+^29^		+^13^^,^^29^		+^13^^,^^29^				
22.	Quality of Life Index (QLI)	Generic					+^13^				
23.	Spine Oncology Study Group Outcomes Questionnaire (SOSG-OQ)	Cancer	+^13^		+^13^						
24.	St Christopher's Index of Patient Priorities (SKIPP)	Generic			+^36^						
25.	Structured Interview for Symptoms and Concerns in PC (SISC)	Cancer		+^37^	+^37^		+^37^	+^37^			
26.	University of Washington Quality of Life Chinese Version (UWQOL-C)	Cancer		+^9^	+^9^		+^9^				
***Care experience***											
1.	Care Evaluation Scale (CES/CES-10)	Cancer		+^21^	+^21^						
2.	Caring Of the Dying Evaluation (CODE)	Generic	+^21^		+^21^						
3.	Chinese Patient Satisfaction Questionnaire (ChPSQ-9)	Cancer	+^9^				+^9^				
4.	Consumer Quality Index Palliative Care (CQ-Index-PC)	Generic			+^21^						
5.	Evaluating Care and Health Outcomes-for the Dying (ECHO-D)	Cancer	+^21^		+^21^	+^21^	+^21^				
6.	End-of-life in Dementia Scales—Satisfaction with Care (SWC-EOLD)	Dementia	+^21^								
7.	European Organisation for Research and Treatment of Cancer (EORTC), Satisfaction with In-Patient Cancer Care–EORTC IN-PATSAT32	Cancer	+^9^								
8.	FATE-32	Generic	+^21^		+^21^						
9.	Family Perceptions of Care Scale (FPCS)	Generic			+^21^	+^21^					
10.	Family satisfaction with end-of-life care (FAMCARE/FAMCARE-5/FAMCARE-10)	Cancer	+^21^			+^21^					
11.	Health Competence Beliefs Inventory (HCBI)^a^	Cancer	+^25^				+^25^				
12.	Postmortem Questionnaire-Short Form (QPM-SF)	Cancer			+^21^	+^21^					
13.	Quality Care Questionnaire- Palliative Care (QCQ-PC)	Cancer	+^9^	+^9^	+^9^						
14.	Quality Measure for Palliative Nursing	Generic			+^36^						
15.	Quality of Communication Questionnaire (QOC)	Generic					+^36^				
16.	Quality of End-of-Life Care (QEOLC)	Generic	+^36^		+^36^		+^36^				
17.	Quality of oncology nursing care scale (QONCS)	Cancer	+^9^	+^9^	+^9^						
18.	Satisfaction Scale for Family members receiving Inpatient Palliative Care (SAT-Fam-IPC)	Generic			+^21^	+^21^					
19.	Sense of Security in Care—Patients' Evaluation (SEC-P)	Generic			+^36^						
***Quality of dying***											
1.	Good Death Inventory (GDI/GDI-sv)	Generic	+^15^				+^15^				
2.	QODD Questionnaire (QODD)	Generic	+^21^^,^^29^		+^29^		+^29^				
	QODD- Korean	Generic	+^15^								
	QODD -Chinese	Generic	+^15^			+^15^					
***Suffering***											
1.	Cancer Distress Scales for Adolescents and Young Adult^a^	Cancer		+^25^	+^25^		+^25^	+^25^	+^25^		
2.	Mini-Suffering State Examination (MSSE)	Generic		+^33^						+^33^	
3.	Patient Dignity Inventory (PDI)	Generic	+^29^		+^13^^,^^38^		+^29^^,^^38^				
4.	Pictorial Representation of Illness and Self-Measure (PRISM)	Generic		+^37^	+^37^		+^37^	+^37^			
5.	State Of Suffering-Five (SOS-V)	Cancer			+^37^		+^37^				
6.	Suffering Assessment Tool (SAT)	Generic			+^37^						
7.	Suffering Scale	Pelvic pain	+^37^								
8.	The Suffering Pictogram	Generic	+^33^		+^33^						
9.	The Suffering Scales	Generic	+^37^	+^37^			+^37^				

Psychometric properties: sufficient ‘+’ See Table A3 for criteria interpretation.

Details on overall grading methodology can be found in the Appendix.

a YA (Young adults).

**Table 5:** Measures with sufficient evidence on psychometric properties and low risk of bias.^a^

#	Measure	Disease group	Internal consistency	Reliability	Content validity	Structural validity	Hypothesis testing	Responsiveness	Cross cultural validity	Criterion validity	Measurement error
***Quality of Life***											
1.	Cancer Module Adolescent Form^b^	Cancer	+^25^								
2.	Generic Core Scales Adolescent Form^b^	Cancer, blood disorders	+^25^				+^25^				
***Care experience***											
1.	Care Evaluation Scale (CES), CES-10	Cancer		+^21^	+^21^						
2.	Caring of the Dying Evaluation (CODE)	Generic	+^21^		+^21^						
3.	Consumer Quality Index Palliative Care (CQ-Index-PC)	Generic			+^21^						
4.	End-of-life in Dementia Scales—Satisfaction with Care (SWC-EOLD)	Dementia	+^21^								
5.	Evaluating Care and Health Outcomes-for the Dying (ECHO-D)	Cancer			+^21^						
6.	FATE-32	Generic			+^21^						
7.	Family Perceptions of Care Scale (FPCS)	Generic			+^21^						
8.	Health Competence Beliefs Inventory (HCBI)^b^	Cancer	+^25^								
9.	Postmortem Questionnaire-Short Form (QPM-SF)	Cancer			+^21^						
10.	Satisfaction Scale for Family members receiving Inpatient Palliative Care (SAT-Fam-IPC)	Generic			+^21^	+^21^					
***Quality of dying***											
1.	Good Death Inventory (GDI/GDI-sv)	Generic	+^15^				+^15^				
2.	QODD- Korean	Generic	+^15^								
	QODD -Chinese	Generic	+^15^			+^15^					
***Suffering***											
1.	Cancer Distress Scales for Adolescents and Young Adult^b^	Cancer						+^25^	+^25^		
2.	The Suffering pictogram	Generic	+^33^		+^33^						

Psychometric properties: sufficient ‘+’ See Table A3 for criteria interpretation.

a This table is based on information from only 4 reviews which reported both psychometric properties and risk of bias assessments. Risk of bias was classified as low if methodological quality was graded as excellent or good based on COSMIN 4-point checklist OR Very good or Adequate based on COSMIN Risk of Bias Checklist.

b YA (Young adults).

Of the nine reviews that assessed psychometric properties (Table 4), only four also evaluated the methodological quality of the included studies.^15^^,^^21^^,^^25^^,^^33^ Three^15^^,^^25^^,^^33^ used the COSMIN Risk of Bias checklist, while one applied the COSMIN 4-point checklist.^21^ Across these four reviews, sufficient evidence with low risk of bias was found for internal consistency in nine, content validity in eight, structural validity and hypothesis testing in two measures, reliability, responsiveness and cross cultural validity in one measure (Table 5, Table A7).

QOL measures (N = 83; Table 2): This included 19 generic and 64 disease-specific measures. The most reviewed generic measure was the Medical Outcomes Study Short-Form surveys (SF-36/SF-12/SF-6D; 122 studies), followed by Palliative Care Outcome Scale (POS), with 10–11 items (56 studies), and the EQ-5D (49 studies). All are multilingual (Table A4), and primarily for patients and/or caregivers, with POS also targeting HCPs. Among 12 unique measures (Table 4) with psychometric data,^38^ POS showed sufficient evidence for four properties.

Among 26 cancer-specific measures, European Organisation for Research and Treatment of Cancer QOL Questionnaire (EORTC QLQ; 15–36 items; 134 studies) was the most reviewed, followed by McGill QOL Questionnaire (MQOL; 8–20 items; 56 studies) and Functional Assessment of Cancer Therapy-General (FACT-G; 18–50 items; 39 studies).

Quality of life assessment scale for gastric cancer patients (QLASTCM- Ga) had sufficient evidence for four properties. EORTC for bone metastases (EORTC QLQBM22) and MQOL showed sufficient evidence for four properties, and EORTC QLQ Core 30 for two properties. FACT-C, FACT-G and Functional Assessment of Anorexia/Cachexia Therapy (FAACT) showed sufficient evidence for one property while FACT-Br showed sufficient evidence for two properties.

For dementia-specific measures (n = 15), four focused on proxies (HCPs and/or informal caregivers), two were patient-only, and three covered proxies and patients. QOL in Alzheimer's Disease (QOL-AD; 13–15 items; 62 studies) and Alzheimer Disease-Related QOL (ADRQL; 24–48 items; 18 studies) were most reviewed. No measure showed sufficient psychometric evidence in any property.

Among measures assessing QOL in renal disease patients (n = 9), Kidney Disease QOL (KDQOL; 36–136 items; 32 studies) was most reviewed in more than ten languages (Table A4). None had sufficient psychometric evidence in any property.

Among heart disease QOL measures (n = 7), three focused on heart failure patients and four focused on transplant patients. Kansas City Cardiomyopathy Questionnaire (KCCQ; 12–23 items; 12 studies) was most reviewed, followed by Minnesota Living with Heart Failure Questionnaire (MLHFQ; 21 items; 9 studies).

For other disease-specific measures (n = 6), none had a low risk of bias in any property. The Multiple Sclerosis Impact Scale (MSIS) had sufficient evidence for four properties.

Care experience measures (n = 49; Table 3): These included 33 generic measures and 16 disease-specific (cancer and dementia) measures.

Among generic measures, 15 were intended for caregivers (two for bereaved caregivers), ten for patients and six included both. The Toolkit After-Death Bereaved Family Member Interview (36–74 items; 13 studies), and Family Assessment of Treatment in End-of-Life (FATE; 12 to 58 items; ten studies from the USA and Netherlands as in Table A5) were most reviewed.

Quality of End-of-Life Care (QEOLC) showed sufficient evidence for three psychometric properties. Care of the Dying Evaluation (CODE) and Sat-Fam-IPC reported sufficient evidence with low risk of bias for two properties.^21^ FATE-32 and FPCS reported sufficient evidence with low risk of bias for one property.

Among cancer-specific measures (n = 13), Family Satisfaction with Advanced Cancer Care (FAMCARE; 5–30 items; 25 studies) was the most reviewed targeting both patients and bereaved caregivers. This was followed by The Care Evaluation Scale (CES/CES-10; 6 studies), designed for bereaved caregivers, which showed sufficient evidence for reliability and content validity with a low risk of bias. Quality of Oncology Nursing Care Scale (QONCS) and Quality Care Questionnaire-Palliative Care (QCQ-PC) showed sufficient evidence for three psychometric properties, but without methodological quality data. Evaluating Care and Health Outcomes-for the Dying (ECHO-D) had sufficient evidence for four psychometric properties.

For dementia-specific measures (n = 3), Satisfaction with Care at End-of-life in Dementia (SWC-EOLD; 10–41 items; 13 studies) designed for informal caregivers, was the most reviewed. It had sufficient evidence for internal consistency with low risk of bias.

QOD measures (n = 15; Table 3): These included nine generic and six disease-specific measures. All nine generic measures targeted caregivers or HCPs. Quality of Dying and Death Questionnaire (QODD; 12 to 48 items; 37 studies) is multilingual and most reviewed (Table A5), followed by Good Death Inventory (GDI; 54 items; 12 studies in Asia) and QOD-Hospice/QOD in Long-Term Care (11–23 items; seven studies). With only one review assessing psychometric properties for risk of bias,^15^ QODD showed sufficient evidence for three properties. GDI and QODD-Chinese had sufficient evidence and low risk of bias for two properties, and QODD-Korean for one property.

Among the six disease-specific measures, three targeted cancer, two targeted renal disease and one targeted dementia. Good Death Scale (GDS; 5 items; 8 studies) was most reviewed for cancer, Dialysis Discontinuation Quality of Dying targeting HCPs (DDQOD; 3 studies) for renal patients and Comfort Assessment in Dying (CAD-EOLD; 13 studies) for dementia patients. None had sufficient psychometric data.

Suffering (n = 14; Table 3): These included nine generic and five disease-specific measures. Among the generic measures, Pictorial Representation of Illness and Self-Measure (PRISM; a non-verbal (visual) measure, available in English, German and Spanish; 1 item; 25 studies; Table A5) and its versions (PRISM-R1/PRISM-R2) were the most reviewed. PRISM, had sufficient evidence for four properties and Patient Dignity Inventory (PDI; 24 studies) and The Suffering Scales for three.

Mini-Suffering State Examination or MSSE (10 items; 8 studies) was originally for those with advanced dementia,^42^ and later validated among patients with advanced cancer.^43^^,^^44^ It had sufficient evidence for two properties. The Suffering Pictogram, another visual measure (eight items) had sufficient evidence and low risk of bias for two properties.

Among disease-specific measures, two were designed for cancer patients, of which State of Suffering-Five (SOS-V; 2 studies, 69 items) had sufficient evidence for two properties, and the Distress Scales for Adolescents and Young Adult (three studies) had sufficient evidence for five properties, of which two were with low risk of bias.

Suffering Scale (1 study, 10 items), focusing on patients experiencing pelvic pain, had sufficient evidence for one property.

## Discussion

Numerous systematic reviews have examined measures used to assess EOL care, but this umbrella review is the first comprehensive effort to systematically synthesize existing reviews and provide an overview of these measures. Most measures focused on QOL at the EOL (52%), followed by care experience (30%), QOD (9%), and suffering (9%), spanning both generic and disease-specific approaches from patients, caregivers, and HCP perspectives. No single measure demonstrated psychometric robustness across all properties, underscoring the lack of a universally recommended tool. Generic measures enable comparisons across diseases and against population norms, making them valuable for broader evaluations, while disease-specific measures capture condition-specific nuances providing a more precise assessment of treatment effects, as supported by the Wilson and Cleary model and subsequent studies.^45^, ^46^, ^47^ Responsiveness, or sensitivity to change, was one of the least assessed psychometric properties, and deserves more attention both for generic and disease specific measures.

In the following discussion, we highlight certain measures based on the specific EOL outcome of interest, target population and respondent, number of items, setting and psychometric properties, and risk of bias. Consistent with established guidelines, we considered content validity to be a critical property for selection.^48^ Users may refer to the checklist in Box 1 as a practical guide to support context-specific measure selection. It does not generate scores or recommend measures but prompts structured reflection. The checklist was developed by the study authors, based on the aspects they deemed relevant when extracting data for this review.

Box 1 **Selecting a measure to assess quality of end-of-life care in your setting**

1. Define your measurement needs–Start by clarifying what you want to measure.
  i. What is the primary outcome you want to measure?
    ☐ Quality of life
    ☐ Care experience
    ☐ Quality of dying
    ☐ Suffering
  ii. What is the target disease group?
    ☐ Generic (applicable to any serious illness)
    ☐ Disease-specific (e.g., cancer, organ failure, dementia) — specify
  iii. Who are the respondents (you may select multiple options)?
    ☐ Patients
    ☐ Caregivers/family members
    ☐ Healthcare professionals
  iv. In what care setting will the tool be used (you may select multiple options)?
    ☐ Community/Home
    ☐ Hospital (inpatient or outpatient)
    ☐ Long-term care facilities/Nursing homes
    ☐ Hospice or palliative care units
  v. What is your preference regarding the minimum and maximum number of items in the measure?

*_Min: ___ Max: ____.*

*(Consider trade-off between comprehensiveness and respondent burden)*.

2. Generate a list of candidate tools from this umbrella review that best fit your measurement needs
3. Evaluate each measure:
For each measure complete the following:
  vi. Has the measure been validated in your country or language of administration? (*refer to* Tables A4, A5)
    ☐ *Yes → Proceed to step vii*
    ☐ *No → Search literature database or consider an alternative tool or cross-cultural adaptation*
  vii. Complete the Measure Evaluation Table below.

**Table:** Measure Evaluation Table

Properties	Measurement properties—Sufficient (Table 4)	Methodological quality—Low risk of bias (Table 5)
Content validity^a^	☐	☐
Internal consistency	☐	☐
Reliability	☐	☐
Structural validity	☐	☐
Hypothesis testing	☐	☐
Responsiveness	☐	☐
Cross-cultural validity	☐	☐
Criterion validity	☐	☐
Measurement error	☐	☐


☐ Measurement properties are acceptable^b^ with low risk of bias	→ **Measure is Recommended**
☐ Measurement properties are acceptable^b^ but risk of bias for evidence is not reported or high	→ **Measure is Recommended with caution** (consider further validation)
☐ Measurement properties are not acceptable^b^ with low risk of bias	→ **Measure is Not recommended**
☐ Measurement properties are not acceptable^b^ but risk of bias for evidence is not reported or high	→ **Measure is Not recommended**

a Content validity must be sufficient before other properties are reviewed.

b Measurement properties are considered acceptable if there is sufficient evidence for the specific measurement properties relevant to the measure's intended use.

For seriously ill patients with diverse diagnoses, POS is commonly used for screening patients for their symptoms, needs or concerns across various settings, but lacked methodological quality assessment in the included reviews.^13^^,^^29^, ^30^, ^31^^,^^36^ For care experience, FATE, CODE and QEOLC are relatively psychometrically robust. FATE, has a shorter 16-item version to reduce respondent burden,^49^^,^^50^ but has only been evaluated within the U.S. Veteran population. CODE demonstrates robust psychometric properties in international contexts beyond its original UK validation.^51^^,^^52^ The 40-item CODE is also a more user-friendly version^51^ of the 91-item ECHO-D, a comprehensive survey assessing best practices in end-of-life care. QEOLC assesses physicians EOL care skills but is less extensively reviewed.^53^ Other promising measures–FPCS and Sat-Fam-IPC are specific to long-term care^54^ and inpatient palliative care settings.^55^^,^^56^

QODD shows promise for QOD assessment, has sufficient content validity and has been widely administered across countries. For suffering, we recommend PRISM, PDI or The Suffering Pictogram. PRISM is widely used in clinical settings, offering a simple visual assessment, but needs validation in non-Western contexts.^57^, ^58^, ^59^, ^60^, ^61^ PDI, assesses dignity-related distress, is also widely used across countries. The Suffering Pictogram was validated in Malaysia.^62^ Future studies should validate PRISM and The Suffering Pictogram in diverse settings and disease groups.

For advanced cancer patients, QOL can be assessed using EORTC, FACIT, and MQOL, with EORTC and FACIT also have versions tailored to specific cancer types. Among care experience measures, CES, QONCS, QCQ-PC or ECHO-D/CODE are relatively psychometrically robust. CES requires testing beyond Japan.^63^^,^^64^ QONCS focuses only on nursing care and QCQ-PC is validated only in Asia (China and Korea).^9^^,^^65^ CODE is preferred over the lengthy ECHO-D (91 items).^66^ SOS-V offers insights into unbearable suffering but is lengthy and requires broader validation beyond Netherlands,^67^ while CDS-AYA is specific to young adults.

For dementia patients, many generic measures are unsuitable. QOL can be assessed using QOL-AD and ADRQ. QOL-AD^6^ is brief, applicable to patients and proxies across all dementia stages, is widely used but lacks evidence on responsiveness, discriminant validity, acceptability, and factor structure.^5^ ADRQL is proxy-reported, evaluates both positive and negative behaviors but lacks a physical functioning domain.^5^^,^^6^ SWC-EOLD is the most common measure assessing family caregivers’ satisfaction with dementia care and demonstrates strong internal consistency but indeterminate content validity, and its methodological quality was not reported. CAD-EOLD, though having indeterminate content validity, assesses QOD by looking at key palliative care outcomes in advanced dementia patients.^68^ MSSE is commonly used to assess suffering among patients with dementia, however it was reported to have insufficient content validity in the included reviews.^42^

For renal patients, none of the measures were validated, however QOL can be assessed using KDQOL. KDQOL is widely used but lacks items on cognitive function, sleep, and finances.^7^ Its methodological quality remains inconsistent, and further assessment is needed.^4^^,^^7^ DDQOD, a proxy-reported QOD measure, has only been reported in three studies, does not assess pain and distress following dialysis termination, and requires refinement and broader validation.^69^

Similarly, for heart disease while KCCQ and MLHFQ are widely used QOL measures, they lack evidence of validation in seriously ill patients. KCCQ is more predictive of mortality, transplant need, and hospitalization.^70^ No measures assessed care experience or suffering in renal and heart disease patients, nor QOD in heart disease patients.

Our review has limitations. Findings are constrained by the quality, accuracy and comprehensiveness of the included reviews. Most reviews failed two critical AMSTAR2 domains—protocol registration and methodological quality assessment. Some reviews omitted key tool characteristics or assessed only specific versions, while others focused on a few properties. Notably, only nine reviews followed COSMIN guidelines to assess psychometric properties, highlighting a major gap in review methodology. We did not reappraise the original primary studies using the latest COSMIN criteria; rather, we synthesized the psychometric assessments reported in the included reviews, which predominantly relied on earlier COSMIN versions. Therefore, our synthesis may not fully reflect the updates introduced in the most recent COSMIN guidance. Further, the inconsistency in how psychometrics were evaluated using variations of the COSMIN guidelines^21^^,^^37^^,^^41^^,^^71^ further complicates interpretation. To address this, we applied a unified minimum criterion for rating each psychometric property as ‘sufficient’. Additionally, measures commonly used in clinical practice or research may have been overlooked if they were not synthesized in previous systematic reviews. We also acknowledge that the distinction between QOL, care experience, QOD, and suffering is not always clear, and that alternative interpretations are possible. A further limitation of our approach is the omission of widely used symptom assessment measures such as the Edmonton Symptom Assessment Scale. Although these symptom assessment measures are extensively used and may include global or multidimensional items, they were not included as QOL measures based on our criteria. Readers should interpret our findings with this boundary in mind.

Despite these limitations, this review identifies critical gaps for future research. First, to ensure robust measurement, measures must demonstrate good psychometric properties, with content validity being essential. They should have a low risk of bias and be appropriately targeted to the right populations.^72^ However, many EOL measures lacked sufficient evidence for key properties such as content validity, cross-cultural validity, test-retest reliability, and responsiveness—properties essential for evaluating palliative care interventions. Thus, rather than developing new generic measures, improving and validating existing measures using the latest COSMIN guidelines should be the focus.^28^ Second, development and validation of utility-based QOL measures specifically designed for the EOL context remains an important area of research. While EQ-5D and SF-6D were widely used, they are limited in assessing domains most valued at the EOL. Even utility-based measures derived from disease-specific QoL tools, such as those based on the EORTC QLQ-C30 and FACT-G,^73^^,^^74^ differ from their palliative care counterparts- QLQ-C15-PAL and FACIT-Pal-limiting their applicability in EOL settings despite being specific to the disease context. Capability-based measures, like ICECAP-Supportive Care Measure (not assessed in any systematic reviews included) offer promising alternatives and need further evaluation.^75^ Third, gaps remain in measures assessing care experience, QOD, and suffering in specific disease groups — areas that need targeted development. Fourth, future systematic reviews in the field should apply the latest COSMIN guidelines^76^ to ensure alignment with current best practices. Fifth, underreporting of negative psychometric findings remains a challenge. Measures with poor validity are rarely published, limiting transparency. Publishing nuanced evaluations, including less favorable results, is essential for guiding appropriate measure selection. Lastly, implementation research is needed to inform the practical integration of these tools into routine EOL care across diverse clinical settings.

To conclude, this umbrella review found no single measure demonstrated sufficient psychometric robustness across all domains. The striking lack of validated disease-specific measures for care experience, QOD and suffering in some non-cancer populations, underscores an urgent need to address this gap. To advance the field, future research must prioritize rigorous psychometric evaluations of existing tools across diverse cultural contexts and disease groups. Methodologically sound studies are essential to enhance the accuracy and applicability of these measures. Furthermore, standardization in both validation studies and systematic reviews is imperative, with adherence to established reporting guidelines. Addressing these gaps is not merely an academic pursuit, it is a crucial step toward improving the assessment and ultimately provision of EOL care.

## Contributors

Chetna Malhotra: Conceptualization, Supervision, Writing- Original draft preparation, Reviewing and Editing. Louisa Camille Ranoa Poco: Data curation, Methodology, Writing- Original draft preparation, Reviewing and Editing. Shimoni Shah: Data curation, Methodology, Writing- Original draft preparation, Reviewing and Editing. Rowan Harwood: Conceptualization, Critical review and editing, Jotheeswaran Thiyagarajan: Conceptualization, Critical review and editing, Moise Muzigaba: Conceptualization, Critical review and editing. LRP and SS have verified the underlying data. All authors read and approved the final version of the manuscript.

## Data sharing statement

This study was based on previously published data and is therefore available in the original studies. The study protocol was registered at PROSPERO under the registration ID: PROSPERO CRD42024610359. All data generated in this study are included in this article and its Appendix.

## Declaration of interests

All authors declare that they have no competing interests.
