# Supplementary material for: An umbrella review of patient- and carer-reported measures for assessing adult end-of-life care quality outcomes
Source: eClinicalMedicine. 2025 Sep 20;89:103516. doi: 10.1016/j.eclinm.2025.103516 (PMC12495440; doi:10.1016/j.eclinm.2025.103516)
Supplement: Supplementary Tables [file mmc1.docx]

**Supplementary material**

**Search Strategy**

Database: *Medline (PubMed)*

(((((("Systematic Review"[Publication Type:noexp] OR "Systematic Reviews as Topic"[MeSH Terms:noexp] OR "Cochrane Database Syst Rev"[Journal] OR "evid rep technol assess full rep"[Journal] OR "evid rep technol assess summ"[Journal] OR "systematic"[Title]) OR ("systematic search"[Title/Abstract:~1] OR "systematic searches"[Title/Abstract:~1] OR "systematically searched"[Title/Abstract:~1]) AND ("databases"[Title/Abstract] OR "cinahl"[Title/Abstract] OR "cochrane"[Title/Abstract] OR "embase"[Title/Abstract] OR "psycinfo"[Title/Abstract] OR "pubmed"[Title/Abstract] OR "medline"[Title/Abstract] OR "scopus"[Title/Abstract] OR "web science"[Title/Abstract:~1] OR (("electronic database"[Title/Abstract:~1] OR "electronic databases"[Title/Abstract:~1] OR "databases searched"[Title/Abstract:~3]) AND ("eligibility"[Title/Abstract] OR "excluded"[Title/Abstract] OR "exclusion"[Title/Abstract] OR "included"[Title/Abstract] OR "inclusion"[Title/Abstract])) AND ("meta-analysis"[Title/Abstract] OR "systematic"[Title/Abstract])) OR ("evidence assessment"[Title/Abstract] AND "GRADE"[Title/Abstract]) OR ("PRISMA"[Title/Abstract] AND ("guideline"[Title/Abstract] OR "guidelines"[Title/Abstract] OR "preferred"[Title/Abstract] OR "reporting"[Title/Abstract] OR "requirements"[Title/Abstract])) OR "PRISMA-P"[Title/Abstract:~0] OR "systematic psychometric review"[Title/Abstract:~1] OR "systematic evidence map"[Title/Abstract] OR "systematic mapping"[Title/Abstract:~2] OR "systematic literature"[Title/Abstract:~1] OR "systematic Medline"[Title/Abstract:~2] OR "systematic PubMed"[Title/Abstract:~2] OR "Systematic Review"[Title/Abstract:~2] OR "systematic reviews"[Title/Abstract:~2] OR "systematical review"[Title/Abstract:~1] OR "systematical reviews"[Title/Abstract:~2] OR "systematically identified"[Title/Abstract:~1] OR "systematically review"[Title/Abstract:~1] OR "systematically reviewed"[Title/Abstract:~1] OR "systematized review"[Title/Abstract:~1] AND (((((((((((((Quality of Health Care[MeSH Terms]) OR (Quality of Life[MeSH Terms])) OR (Terminal Care[MeSH Terms])) OR (Palliative Care[MeSH Terms])) OR (Hospice Care[MeSH Terms])) OR (satis* with care*[Title/Abstract])) OR (good death*[Title/Abstract])) OR (bad death*[Title/Abstract])) OR (quality of end-of-life care*[Title/Abstract]) OR (suffering[Title/Abstract]) OR (quality of end-of-life[Title/Abstract])) OR (quality of death*[Title/Abstract]) OR (quality of dying*[Title/Abstract]))) OR (End-of-Life Quality[Title/Abstract])) OR (end-of-life care[Title/Abstract]))) AND ((((((((((Terminally Ill[MeSH Terms]) OR ("Caregivers"[Mesh]) OR (caregiver of patient[Title/Abstract]) OR (end stage*[Title/Abstract])) OR (end-of-life*[Title/Abstract])) OR (hospice*[Title/Abstract])) OR (((advance*[Title/Abstract] OR late[Title/Abstract] OR last[Title/Abstract] OR end[Title/Abstract] OR final) adj1 (stage*[Title/Abstract] OR phase*[Title/Abstract])))) OR (((terminal*[Title/Abstract] OR advance*[Title/Abstract] OR incurable[Title/Abstract] OR life-limit*[Title/Abstract] OR life-threaten*) adj1 (ill*[Title/Abstract] OR disease*[Title/Abstract] OR condition*[Title/Abstract] OR stage*[Title/Abstract])))) OR (dying[Title/Abstract])) OR (Palliative Care[Title/Abstract])))) AND (((((((((((Patient Reported Outcome Measures[MeSH Terms]) OR (outcome assessment[MeSH Terms])) OR (process assessment[MeSH Terms])) OR (Patient Outcome Assessment[MeSH Terms])) OR (assessment tools*[Title/Abstract])) OR (measures of end-of-life care*[Title/Abstract])) OR (End-of-Life Quality Measure*[Title/Abstract])) OR (instrument* measuring*[Title/Abstract])) OR (measurement tools[Title/Abstract]) OR (Assessment tools*[Title/Abstract]))

Database: *CINAHL*

( AB ( end of life care or palliative care or death or dying or terminally ill ) OR AB ( quality of death or good death or quality of dying or good dying or suffering ) ) AND ( AB ( patient reported outcome measures or prom or patient reported outcomes ) OR AB ( instruments or tools or measures or questionnaires or scales ) ) AND ( AB ( terminally ill or terminal care or dying or end of life or palliative care ) OR AB caregiver OR AB (life limiting conditions or life-limiting conditions or life limiting illness or life-limiting illness ) AND (systematic reviews or meta analysis or meta- analysis)

Database: *EMBASE*

('terminally ill patient'/exp OR 'terminal patient' OR 'terminally ill' OR 'terminally ill patient' OR 'dying'/exp OR 'about to die' OR 'approaching death' OR 'close to death' OR 'dying' OR 'dying patient' OR 'moribund' OR 'near death' OR 'patient, dying' OR 'caregiver'/exp OR 'care giver' OR 'caregiver' OR 'caregivers' OR 'carer' OR 'carers' OR 'family caregiver' OR 'family caregivers' OR 'life limiting condition'/exp OR 'life limiting illness'/exp) AND ('patient-reported outcome'/exp OR 'patient reported outcome measures' OR 'patient-reported outcome' OR 'patient-reported treatment outcome' OR 'patientreported outcome' OR 'self-reported outcome' OR 'self-reported patient outcome' OR 'self-reported treatment outcome' OR 'selfreported outcome' OR 'outcome assessment'/exp OR 'health care outcome assessment' OR 'healthcare outcome assessment' OR 'outcome assessment' OR 'outcome assessment (health care)' OR 'outcome assessment, health care' OR 'outcome measurement' OR 'patient outcome assessment' OR 'quality measurement'/exp OR 'instruments measuring':ti,ab OR 'end of life care':ti,ab) AND ('health care quality'/exp OR 'clinical governance' OR 'health care evaluation' OR 'health care evaluation mechanisms' OR 'health care process assessment' OR 'health care quality' OR 'health care quality assurance' OR 'health care quality indicators' OR 'health care quality, access, and evaluation' OR 'healthcare evaluation' OR 'healthcare process assessment' OR 'healthcare quality' OR 'healthcare quality assurance' OR 'healthcare quality indicators' OR 'process assessment (health care)' OR 'process assessment, health care' OR 'quality assurance, health care' OR 'quality indicators, health care' OR 'quality of care research' OR 'quality of health care' OR 'quality of healthcare' OR 'quality, health care' OR 'standard of care' OR 'quality of dying':ti,ab OR 'end of life care':ti,ab OR ‘suffering’:ti,ab) AND ('systematic review':ti,ab OR 'meta analysis':ti,ab)

Database: *Cochrane*

ID Search

#1 (assessment tools* OR measures of end-of-life care* OR End-of-Life Quality Measure* OR (instrument* measuring*) OR measurement tools):ti,ab,kw

#2 (end stage* OR end-of-life* OR hospice* OR ((advance*OR late OR last OR end OR final) adj1 (stage* OR phase*)) OR ((terminal* OR advance*OR incurable OR life-limit*OR life-threaten*) adj1 (ill* OR disease OR condition*OR stage*)) OR dying OR patient-centered OR Palliative Care):ti,ab,kw

#3 MeSH descriptor: [Quality of Health Care] explode all trees

#4 MeSH descriptor: [Quality of Life] explode all trees

#5 MeSH descriptor: [Terminal Care] explode all trees

#6 MeSH descriptor: [Palliative Care] explode all trees

#7 MeSH descriptor: [Hospice Care] explode all trees

#8 MeSH descriptor: [Pain] explode all trees

#9 (satis* with care* OR good death* OR bad death* OR quality of end-of-life care* OR quality of end-of-life OR quality of death* OR quality of dying*OR End-of-Life Quality OR end-of-life care OR suffering):ti,ab,kw

#10 MeSH descriptor: [Terminally Ill] explode all trees

#11 MeSH descriptor: [Patient Reported Outcome Measures] explode all trees

#12 MeSH descriptor: [Outcome Assessment, Health Care] explode all trees

#13 MeSH descriptor: [Process Assessment, Health Care] explode all trees

#14 MeSH descriptor: [Patient Outcome Assessment] explode all trees

#15 (#3 OR #4 OR #5 OR #6 OR #7 ) AND (#10 OR #2 ) AND (#11 OR #12 OR #13 OR #14 OR #1 )

#16 MeSH descriptor: [Caregivers] explode all trees

#17 (caregivers):ti,ab,kw

#18 (#3 OR #4 OR #5 OR #6 OR #7 OR #8 OR #9) AND (#10 OR #2 OR #16 OR #17) AND (#11 OR #12 OR #13 OR #14 OR #1 )

Database: *SCOPUS*

(TITLE-ABS((quality of healthcare) OR (quality of care when dying) OR (terminal care) OR (palliative care) OR (hospice care) OR (satisfaction with care*) OR (good death*) OR (bad death*) OR (quality of end-of-life*) OR (quality of death*) OR (quality of dying*)) AND TITLE-ABS-KEY( (terminally ill patient) OR (caregiver of terminally ill patient) OR (caregiver) OR (caregiver of dying patient) OR (end stage*) OR (end-of-life*) OR (hospice*) OR ((advance* OR late OR last OR end OR final) AND (stage* OR phase*)) OR ((terminal* OR advance* OR incurable OR life-limit* OR life-threaten*) AND (ill* OR disease* OR condition* OR stage*)) OR (dying) OR (suffering) OR (palliative care)) AND TITLE-ABS( (patient reported outcome measure) OR (outcome assessment) OR (process assessment) OR (assessment tool*) OR (measures of end-of-life care*) OR (End-of-life quality measure*) OR (instrument* measuring* ) OR (measurement tools) ) AND TITLE-ABS((systematic* W/3 (review* OR overview* ))) OR TITLE-ABS("data synthes*" OR "data extraction*" OR "data abstraction*" ) OR TITLE-ABS("met analy*" OR metanaly*) OR TITLE-ABS("meta regression*" OR metaregression* ) OR TITLE-ABS(meta-analy* OR metaanaly* OR "systematic review*" ) OR TITLE-ABS-KEY(medline OR cochrane OR pubmed OR medlars OR embase OR cinahl ) OR TITLE-ABS(multi* W/3 treatment W/3 comparison* ) OR TITLE-ABS(mixed W/3 treatment W/3 (meta-analy* OR metaanaly* )) OR TITLE-ABS(multi* W/2 paramet* W/2 evidence W/2 synthesis ) OR TITLE-ABS(multiparamet* W/2 evidence W/2 synthesis ) OR TITLE-ABS(multi-paramet* W/2 evidence W/2 synthesis))

**Table A1: Conceptual framework of the four focal categories in quality of end-of-life care**

|  | **Quality of Life** | **Care experience** | **Quality of Dying** | **Suffering** |
| --- | --- | --- | --- | --- |
| Working Definitions | How individuals perceive, evaluate, and articulate their lived experience across multiple domains of well-being while living with serious illness. This includes both subjective evaluations of well-being and the identification of unmet needs that may impact overall quality of life. | Evaluation of perceptions, experiences, or satisfaction with the care received | An extension of quality of life that focuses specifically on the dying phase (final days to weeks), | Captures the individual’s severe distress - physical, psychological, existential, or spiritual- especially when threatened in their personhood, dignity, or integrity. |
| Respondents | Primarily self-report; proxy-report used when patients are unable (e.g. in advanced dementia). | Patient, caregiver, or bereaved family member; often caregiver proxy post-death. | Mostly caregiver or clinician proxy (after death). | Mostly patient self-report; sometimes inferred by clinician or family. |
| Time orientation | During illness trajectory. | During illness or post-death (bereavement). | Typically retrospective- final days, weeks, or month of life. | During illness or dying |
| Example domains included in measurement scales | Physical well-being (e.g. pain, fatigue, mobility, sleep, appetite); Psychological or emotional well-being (coping);  Social well-being (social support, roles, interactions, interpersonal relationships, sexual function); Functional well-being (independence, ADLs/IADLs, ability to work); Existential or spiritual well-being (meaning, peace, hope); Overall life-appraisal; Identified needs or unmet needs (Practical, informational, emotional, and spiritual needs that require support or intervention). | Communication quality (clarity, honesty, shared decision-making);  Respect, dignity and compassion (cultural sensitivity); Responsiveness to needs and preferences; Continuity and coordination of care, (care planning, discharge planning, team integration);  Family involvement and support; Access to care and information (timely information, access to services, timeliness of care);  Appropriateness of care; satisfaction with care (overall ratings, willingness to recommend care) | Physical comfort (symptom management),  Psychological comfort (freedom from fear, emotional closure),  Spiritual/existential peace (sense of completion, religious rituals); Dignity and respect (maintaining identity, privacy);  Preparedness and life closure (goodbyes, sense of completion, life review); Concordance with preferences (preferred place and manner of dying); Family presence and support; Awareness, acceptance and control  (over dying process; autonomy of choices); cultural and personal rituals | Existential distress (loss of meaning); Psychological anguish (fear, hopelessness, anxiety); Physical suffering (unrelieved symptoms); Loss of meaning or purpose; Social disconnection (isolation, abandonment); Loss of dignity and personhood; Identity disruption (loss of roles, image, control); spiritual suffering (loss of faith) |

**Table A2. AMSTAR Grading of reviews**

| **Review** | **AMSTAR2 checklist** | | | | | | | | | | | | | | | |
| --- | --- | --- | --- | --- | --- | --- | --- | --- | --- | --- | --- | --- | --- | --- | --- | --- |
| Author, year of publication | 1  Did the research questions and inclusion criteria for the review include the components of PICO? | 2  Did the report of the review contain an explicit statement that the review methods were established prior to the conduct of the review and did the report justify any significant deviations from the protocol? | 3  Did the review authors explain their selection of the study designs for inclusion in the review? | 4  Did the review authors use a comprehensive literature search strategy? | 5  Did the review authors perform study selection in duplicate? | 6  Did the review authors perform data extraction in duplicate? | 7  Did the review authors provide a list of excluded studies and justify the exclusions? | 8  Did the review authors describe the included studies in adequate detail? | 9  Did the review authors use a satisfactory technique for assessing the risk of bias (RoB) in individual studies that were included in the review? | 10  Did the review authors report on the sources of funding for the studies included in the review? | 11  If meta-analysis was performed did the review authors use appropriate methods for statistical combination of results? | 12  If meta-analysis was performed, did the review authors assess the potential impact of RoB in individual studies on the results of the meta-analysis or other evidence synthesis? | 13  Did the review authors account for RoB in individual studies when interpreting/discussing the results of the review? | 14  Did the review authors provide a satisfactory explanation for, and discussion of, any heterogeneity observed in the results of the review? | 15  If they performed quantitative synthesis did the review authors carry out an adequate investigation of publication bias (small study bias) and discuss its likely impact on the results of the review? | 16  Did the review authors report any potential sources of conflict of interest, including any funding they received for conducting the review? |
| Aiyegbusi et al. 2017 | Yes | Yes | Yes | Yes | Yes | Yes | Partial Yes | Yes | Yes | Yes | No meta-analysis conducted | No meta-analysis conducted | Yes | Yes | No meta-analysis conducted | Yes |
| Albers et al. 2010 | No | No | Yes | Yes | No | Yes | No | Partial Yes | No | No | No meta-analysis conducted | No meta-analysis conducted | No | Yes | No meta-analysis conducted | No |
| Bausewein et al. 2011 | No | No | Yes | Partial Yes | No | No | Partial Yes | Yes | No | Yes | No meta-analysis conducted | No meta-analysis conducted | No | Yes | No meta-analysis conducted | Yes |
| Bowling et al. 2015 | Yes | No | Yes | Yes | Yes | No | Partial Yes | Yes | No | Yes | No meta-analysis conducted | No meta-analysis conducted | No | Yes | No meta-analysis conducted | Yes |
| Burks et al. 2021 | Yes | No | Yes | Partial Yes | Yes | No | Partial Yes | Partial Yes | Yes | Yes | No meta-analysis conducted | No meta-analysis conducted | Yes | Yes | No meta-analysis conducted | Yes |
| Chambers et al 2025 | Yes | Yes | Yes | Yes | No | Yes | No | Yes | No | Yes | No meta-analysis conducted | No meta-analysis conducted | No | Yes | No meta-analysis conducted | Yes |
| Correia et al. 2011 | No | No | No | Partial Yes | No | No | No | Partial Yes | No | No | No meta-analysis conducted | No meta-analysis conducted | No | No | No meta-analysis conducted | No |
| Ferreira et al 2025 | Yes | No | Yes | Yes | Yes | Yes | No | Yes | Yes | Yes | No meta-analysis conducted | No meta-analysis conducted | Yes | Yes | No meta-analysis conducted | Yes |
| Glover et al. 2011 | No | No | Yes | No | No | No | No | No | No | Yes | No meta-analysis conducted | No meta-analysis conducted | No | Yes | No meta-analysis conducted | Yes |
| Gutierrez Sanchez et al. 2018 | Yes | Yes | Yes | Partial Yes | Yes | Yes | Partial Yes | Yes | No | No | No meta-analysis conducted | No meta-analysis conducted | No | Yes | No meta-analysis conducted | Yes |
| Gutierrez-Sanchez et al. 2020 | Yes | Partial Yes | Yes | Yes | Yes | Yes | Yes | Yes | Yes | Yes | No meta-analysis conducted | No meta-analysis conducted | Yes | Yes | No meta-analysis conducted | Yes |
| Hales et al. 2010 | No | No | Yes | Partial Yes | Yes | No | No | Partial Yes | No | No | No meta-analysis conducted | No meta-analysis conducted | No | Yes | No meta-analysis conducted | Yes |
| Holden et al. 2015 | No | No | Yes | No | No | No | No | No | No | Yes | No meta-analysis conducted | No meta-analysis conducted | No | Yes | No meta-analysis conducted | Yes |
| Hounsome et al. 2011 | Yes | No | Yes | Partial Yes | No | No | No | Yes | No | No | No meta-analysis conducted | No meta-analysis conducted | No | Yes | No meta-analysis conducted | No |
| Kearns et al. 2017 | Yes | No | Yes | Yes | No | No | Partial Yes | Partial Yes | No | Yes | No meta-analysis conducted | No meta-analysis conducted | No | Yes | No meta-analysis conducted | Yes |
| Krikorian et al. 2013 | Yes | No | Yes | Partial Yes | Yes | No | No | Yes | No | Yes | No meta-analysis conducted | No meta-analysis conducted | No | Yes | No meta-analysis conducted | Yes |
| Kupeli et al. 2019 | Yes | Yes | Yes | Yes | No | Yes | Partial Yes | Partial Yes | No | Yes | No meta-analysis conducted | No meta-analysis conducted | No | Yes | No meta-analysis conducted | Yes |
| Lam et al. 2022 | No | No | Yes | Yes | Yes | Yes | Partial Yes | Yes | Yes | No | No meta-analysis conducted | No meta-analysis conducted | Yes | Yes | No meta-analysis conducted | Yes |
| Lendon et al. 2015 | Yes | No | Yes | Yes | Yes | Yes | No | Partial Yes | No | Yes | No meta-analysis conducted | No meta-analysis conducted | No | Yes | No meta-analysis conducted | Yes |
| Li et al. 2023 | Yes | No | Yes | Yes | No | No | Partial Yes | Yes | No | Yes | No meta-analysis conducted | No meta-analysis conducted | No | Yes | No meta-analysis conducted | Yes |
| Li et al. 2024 | Yes | Yes | Yes | Yes | Yes | Yes | Partial Yes | Partial Yes | Yes | Yes | No meta-analysis conducted | No meta-analysis conducted | Yes | Yes | No meta-analysis conducted | Yes |
| Mahmoudi et al. 2022 | No | No | Yes | No | Yes | No | No | Yes | No | Yes | No meta-analysis conducted | No meta-analysis conducted | No | Yes | No meta-analysis conducted | Yes |
| Peng et al, 2019 | Yes | No | Yes | Yes | No | No | Partial Yes | Partial Yes | Yes | Yes | Yes | Yes | No | Yes | No | Yes |
| Potts et al. 2018 | Yes | No | Yes | Yes | No | No | No | Yes | No | Yes | No meta-analysis conducted | No meta-analysis conducted | No | Yes | No meta-analysis conducted | Yes |
| Quigley et al. 2020 | Yes | No | No | Yes | Yes | Yes | Partial Yes | Partial Yes | No | Yes | No meta-analysis conducted | No meta-analysis conducted | No | Yes | No meta-analysis conducted | Yes |
| Roydhouse et al. 2017 | Yes | No | Yes | Partial Yes | No | No | Partial Yes | Yes | No | Yes | No meta-analysis conducted | No meta-analysis conducted | No | Yes | No meta-analysis conducted | Yes |
| Stiel et al. 2012 | Yes | No | Yes | Yes | No | Yes | No | No | No | Yes | No meta-analysis conducted | No meta-analysis conducted | No | Yes | No meta-analysis conducted | Yes |
| van Roij et al. 2018 | Yes | No | Yes | Yes | Yes | Yes | Partial Yes | Yes | No | Yes | No meta-analysis conducted | No meta-analysis conducted | No | Yes | No meta-analysis conducted | Yes |
| Virdun et al. 2023 | Yes | No | Yes | Yes | No | No | Partial Yes | Yes | No | Yes | No meta-analysis conducted | No meta-analysis conducted | No | Yes | No meta-analysis conducted | Yes |
| Xu et al. 2023 | Yes | Yes | Yes | Yes | Yes | No | Partial Yes | Yes | Yes | Yes | No meta-analysis conducted | No meta-analysis conducted | Yes | Yes | No meta-analysis conducted | Yes |

Critical domains

**Table A3. Criteria for Rating Psychometric Properties as Sufficient: From Included Systematic Reviews and the Unified Thresholds for Cross-Comparison**

| **Criteria versions** | **Kupeli et al. 2019^(1)^** | **Krikorian et al. 2011^(2)^** (following Bot et al. 2003)**^(3)^** | **Terwee et al. 2007^(4)^**  Albers et al. 2010^(5)^, Kearns et al. 2017^(6)^, van Roij et al. 2018^(7)^, Chambers et al. 2025^(8)^, Li et al. 2023^(9)^ | **Prinsen et al. 2018^(10)^**  Gutierrez-Sanchez et al. 2020^(11)^, Xu et al. 2023^(12)^ | **Unified Minimum Criteria** |
| --- | --- | --- | --- | --- | --- |
| **Psychometric Properties** | **Sufficient (Positive in the original criteria)** | **Sufficient (Positive in the original criteria)** | **Sufficient (Positive in the original criteria)** | **Sufficient** | **Sufficient** |
| **Internal consistency** | Data from adequate sample used to conduct factor analysis and Cronbach’s alpha per factor between 0·70 and 0·90 | Adequate design and method; factor analysis supporting the dimension; Cronbach’s alpha > 0·70 for each dimension / subscale | Factor analyses performed on adequate sample size (7 times # items and ≥100) AND Cronbach’s alpha(s) calculated per dimension AND Cronbach’s alpha(s) between 0·70 and 0·95 | At least low evidence for sufficient structural validity AND Cronbach’s alpha(s) ≥ 0·70 for each  unidimensional scale or subscale | Factor analysis performed on adequate sample size (OR at least low evidence for sufficient structural validity) AND Cronbach’s alpha ≥ 0·70-0·90. (Note: Cronbach’s alpha > 0·90 are considered for Prinsen et al. 2018 and Krikorian et al. 2011; and > 0·95 for Terwee et al. 2007) |
| **Reliability** | Intraclass Correlation Coefficient (ICC) or weighted kappa ≥ 0·70 | Adequate design and method AND ICC > 0·70 AND time interval and confidence intervals (or n >50) were presented | ICC or weighted Kappa>0·70* | ICC or weighted Kappa ≥ 0·70 | ICC or (weighted) Kappa ≥0·70 |
| **Content validity** | Detailed description of tool development, including tool aim, target population, concepts under assessment, item selection, and the population (patient and experts) involved in item selection | Patients and investigator/ expert involved during item selection and/or item reduction AND Patients were consulted for reading and comprehension. | A clear description is provided of the measurement aim, the target population, the concepts that are being measured, and the item selection AND target population and (investigators OR experts) were involved in item selection | Included items are relevant for the construct, target population and for the context of use of interest, response options and recall period are appropriate AND all key concepts are included AND Patient-reported Outcome Measure (PROM) instructions, PROM items and response options are understood by the population of interest as intended, PROM items are appropriately worded, and response options match the questions^(13)^ | Description of tool development, including tool aim, target population, concepts under assessment, item selection, and the target population and (experts OR investigators) involved in item selection OR (Included items are relevant for the construct, target population and for the context of use of interest, response options and recall period are appropriate AND all key concepts are included AND PROM instructions, PROM items and response options are understood by the population of interest as intended, PROM items are appropriately worded, and response options match the questions) |
| **Structural validity** | Factor analysis demonstrates that combined set of factors explain ≥ 50% of total variance in the model OR Item Response Theory **(**IRT) methods confirm (uni) dimensionality |  |  | **Classical Test Theory (CTT)**  Confirmatory Factor Analysis (CFA): Comparative Fit Index (CFI) or Tucker-Lewis Index (TLI) or comparable measure > 0·95 OR Root Mean Square Error of Approximation (RMSEA) < 0·06 OR Standardized Root Mean Residuals (SRMR) < 0·08  **IRT/Rasch**  No violation of uni-dimensionality: CFI or TLI or comparable measure > 0·95 OR RMSEA < 0·06 OR SRMR < 0·08  AND no violation of local independence: residual correlations among the items after controlling for the dominant factor < 0.20 OR Q3’s < 0·37 AND no violation of monotonicity: adequate looking graphs OR item scalability > 0·30 AND  adequate model fit IRT: χ 2 > 0·001  Rasch: infit and outfit mean squares ≥ 0·5 and ≤ 1·5 OR Z-standardized values > −2 and < 2 | Factor analysis demonstrates that combined set of factors explain ≥ 50% of total variance in the model OR  **CTT**  CFA: CFI or Tucker-Lewis Index or comparable measure > 0·95 OR RMSEA < 0·06 OR SRMR < 0·08  **IRT/Rasch**: No violation of uni-dimensionality  CFI or TLI or comparable measure > 0·95 OR RMSEA < 0·06 OR SRMR < 0·08 AND no violation of local independence: residual correlations among the items after controlling for the dominant factor < 0·20 OR Q3’s < 0·37 AND no violation of monotonicity: adequate looking graphs OR item scalability > 0·30 AND  adequate model fit  IRT: χ 2 > 0·001  Rasch: infit and outfit mean squares ≥ 0·5 and ≤ 1·5 OR Z-standardized values > −2 and < 2 |
| **Hypothesis testing for construct validity** | Specific hypotheses were formulated and at least 75% of the results in line with hypotheses | Hypotheses were formulated, results were acceptable in accordance with ≥ 75% of hypotheses and adequate measure was used AND adequate design and methods | Specific hypotheses were formulated AND at least 75% of the results are in accordance with these hypotheses | The result is in accordance with the hypothesis (The results of all studies should be taken together and it should then be decided if 75% of the results are in accordance with the hypotheses) | ≥75% of the results is in accordance with predefined hypotheses |
| **Responsiveness** |  | For evaluative questionnaires, responsiveness should be assessed AND hypotheses were formulated, and results were in agreement with ≥ 75% of hypotheses AND an adequate measure was used (effect size, standardized response mean, comparison with external standard) AND adequate design and methods | Smallest Detectable Change (SDC) < Minimal Important Change (MIC) OR MIC outside the Limits of Agreement (LOA) OR Responsiveness Ratio (RR)>1.96 OR Area under the receiver operating characteristics (ROC) curve (AUC) ≥ 0·70*** | The result is in accordance with the hypothesis OR AUC ≥ 0·70 | ≥75% of the results is in accordance with predefined hypotheses OR AUC ≥ 0·70 OR SDC < MIC OR MIC outside the LOA OR RR >1·96. |
| **Cross cultural validity or measurement invariance** |  |  |  | No important differences found between group factors (such as age, gender, language) in multiple group factor analysis OR no important differential item functioning (DIF) for group factors (McFadden’s R2 < 0·02) | No important differences found between group factors (such as age, gender, language) in multiple group factor analysis OR no important DIF for group factors (McFadden’s R2 <0·02) |
| **Criterion validity** |  |  | Convincing arguments that gold standard is “gold” AND correlation with gold standard >0·70** | Correlation with gold standard ≥ 0.70 OR area under the curve (AUC) ≥0·70 | Correlation with gold standard ≥ 0.70 OR area under the curve ≥ 0·70 |
| **Reproducibility (agreement is absolute measurement error); Measurement error** |  |  | SDC < MIC OR MIC outside the limits of agreement (LOA) OR convincing arguments that agreement is acceptable** | SDC or LOA < MIC | SDC or LOA < MIC OR MIC outside LOA OR convincing arguments that agreement is acceptable |

*For Albers et al. 2010 and van Roij et al. 2018 sufficiency includes AND time interval at least 1 week.

** For Li et al. 2023 and Kearns et al. 2017, these properties were not assessed.

*** For van Roij et al. 2018 responsiveness property requires that specific hypotheses were formulated AND at least 75% of the results are in accordance with these hypotheses AND at least two measurements are available AND time interval is described AND SDC < MIC OR MIC outside LOA or RR > 1·96 OR ROC ≥ 0·70

**Table A4. Grading of Methodological Quality for Each Property**

| **Low Risk of Bias** | **COSMIN 4-point checklist**  Kupeli et al. 2019^(1)^ | **COSMIN Risk of Bias checklist**  Chambers et al. 2025^(8)^, Xu et al. 2023^(12)^, Gutierrez-Sanchez et al. 2020^(11)^ |
| --- | --- | --- |
| Yes | Excellent or Good rating | Very good or Adequate rating |
| No | Poor or Fair rating | Inadequate or Doubtful rating |
| Inconclusive | If at the review level, the tool was evaluated more than once for methodological quality, but the ratings are conflicting leading to a tie – i.e., Yes for the same tool in one study, No for the same tool in another study. The rating is stated as inconclusive. | |

**Table A5. Languages and Countries for quality-of-life measures**

| **#** | **Measure** | **Languages** | **Countries** |
| --- | --- | --- | --- |
|  | Affect and Activity Ratings^(14)^ | English | USA |
|  | African Palliative Care Outcome Scale (African POS)^(6, 15, 16)^ |  | Africa, South Africa, Zimbabwe, Tanzania, Kenya, Malawi |
|  | Alzheimer Disease-Related Quality of Life (ADRQL)^(14, 17)^ | English, Japanese | USA, Greece, Australia, US, Germany, Belgium, Japan |
|  | Assessment of Quality of life at the EOL (AQOL)^(7)^ |  |  |
|  | Bath assessment of subjective quality of life in dementia (BASQID)^(14)^ | English |  |
|  | Brain Symptom and Impact Questionnaire (BASIQ)^(7)^ |  |  |
|  | Brief Hospice Inventory (BHI)^(5, 6)^ | English | USA |
|  | Cambridge Palliative Audit Schedule (CAMPAS-R)^(5)^ |  |  |
|  | Cancer Module Adolescent Form^(8)^ | English | Australia |
|  | Chinese Dialysis Quality of Life Scale (CDQOL)^(18)^ | Cantonese | Hong Kong, China |
|  | CHOICE Health Experience Questionnaire (CHEQ)^(18)^ | English, Thai | USA, Thailand |
|  | Chronic Heart Failure Questionnaire (CHFQ)^(19)^ | English, Chinese | Canada, Hong Kong |
|  | Chronic Liver Disease Questionnaire^(20)^ | English | China, USA, Germany, Spain, Mexico, UK, Pakistan, Lithuania, Poland |
|  | Chronic Respiratory Questionnaire (CRQ)^(19)^ | English | Canada |
|  | City of Hope Quality of Life Survey^(16)^ |  | India |
|  | Client Generated Index (CGI)^(21)^ | English | Australia |
|  | Cornell-Brown Scale for Quality of Life (CBS)^(14)^ | English |  |
|  | Darthmouth Cooperative functional health assessment chart and World Organization of General Practice/Family Physicians (COOP/WONCA)^(22)^ |  | Netherlands |
|  | Dementia Quality of Life (DQOL)^(14)^ | English, Chinese, French, Spanish, German | USA, Netherlands, UK, Taiwan, France, Switzerland, Spain, Germany |
|  | DEMQOL/DEMQOL-Proxy^(14, 17)^ | English | USA, UK, Ireland, France, Greece, Poland, Italy, Finland |
|  | End-Stage Renal Disease Symptom Checklist (ESRD-SCL-TM)^(23)^ | Turkish | Turkey |
|  | EORTC Quality of Life Questionnaire^(5, 7, 9, 15, 22, 24)^ | English | Mainland China, UK, Sweden, USA, Netherlands, Canada, Australia, Austria, Sweden |
|  | EQ-5D^(7, 18-20, 22-26)^ | Dutch, French, Multiple languages | UK, Spain, Belgium, Netherlands, China, Singapore, Canada, USA, Sweden, Denmark, Finland, Norway, UK, France |
|  | Ferrans and Powers QOL Index (QLI 3.0)^(8, 23)^ |  |  |
|  | Functional Assessment of Chronic Illness Therapy-Palliative Care (FACIT-Pal)^(5, 7, 15, 19)^ | English | USA |
|  | Functional Assessment of Chronic Illness Therapy (FACT-G/FACIT-Sp/FACT-C/FACT-Br/FAACT)^(5, 7, 8, 15, 19, 22, 24)^ | English, Chinese | Hong Kong, USA, Canada, UK, Japan |
|  | Gastrointestinal Quality of Life Index (GIQLI)^(18)^ | German, English | Australia, Canada, Germany, Switzerland |
|  | Generic Core Scales Adolescent Form^(8)^ | English | Australia |
|  | Health Related Quality of Life^(24)^ |  |  |
|  | Heart Transplant Intervention Scale^(26)^ |  |  |
|  | Heart Transplant Stressor Scale (Jalowiec Stressor Scale) ^(26)^ |  |  |
|  | Hebrew Rehabilitation Centre for Aged Quality of Life (HRCA-QL)^(15, 24)^ | Spanish | Spain |
|  | Hepatitis Quality of Life Questionnaire (HQLQ)^(20)^ | English | Canada |
|  | Hospice Quality of Life Index (HQLI)^(5, 7, 22, 24)^ | English | USA |
|  | Hospice Quality of Life Scale (HQLS)^(15)^ |  | Korea |
|  | Kansas City Cardiomyopathy Questionnaire (KCCQ/KCCQ-12)^(19, 26)^ | English | USA |
|  | Kidney Disease Quality of Life (KDQOL/KDQOL-SF/KDQOL-36)^(18, 23)^ | Arabic, Chinese, Dutch, Danish, English, Farsi, Filipino, French, Greek, Hungarian, Italian, Japanese, Korean, Moroccan, Portuguese, Persian, Russian, Turkish, Taiwanese, Cantonese, Kannada, Spanish, Mandarin, Thai | Egypt, Hungary, Philippines, France, Morocco, Singapore, Brazil, Iran, Japan, USA, Italy, Greece, Netherlands, Denmark, Korea, Switzerland, Russia, Turkey, Taiwan, China, India, Thailand |
|  | Kidney Transplant Questionnaire (KTQ)^(18)^ | English, Chinese, Spanish | USA, Canada, China, Spain |
|  | Life Satisfaction Index (LSI)^(23)^ |  |  |
|  | Linear Analog Scale Assessments (LASAs)^(7)^ |  |  |
|  | Liver Disease Quality of Life Questionnaire (LDQOL)^(20)^ | English | USA |
|  | Lung Cancer Symptom Scale (LCSS)^(15)^ | English | USA |
|  | McGill Quality of Life Questionnaire (MQOL/MQOL-HK/CSF)^(5, 7, 9, 15, 22, 24)^ | Chinese, Korean, Japanese, Hebrew | Malaysia, Mainland China, Taiwan, Canada, Israel, Hong Kong, Korea, Japan |
|  | McMaster Quality of Life Scale (MQLS)^(5, 24)^ |  |  |
|  | Medical Outcomes Study Short Form (SF-36/SF-36v2/SF-12/SF-6D)^(8, 18-20, 22-24, 26)^ | English, Italian, Persian, Multiple languages | USA, Italy, China, Germany, Sweden, Spain, Brazil, Switzerland, Netherlands, Canada, India, UK, Singapore, Austria, Poland, Portugal, Iran |
|  | Minnesota Living with Heart Failure Questionnaire (MLHFQ)^(19, 26)^ | English | USA |
|  | Missoula-VITAS Quality of Life Index (MVQOLI/MVQOLI-R)^(5-7, 15, 24)^ | English | South Africa, Uganda, USA |
|  | Modified Chinese QoL questionnaire^(20)^ |  | China |
|  | Modified City of Hope Patient Questionnaire (MCOHPQ)^(6)^ | English | USA |
|  | Multiple Sclerosis Impact Scale (MSIS)^(6)^ | English | UK |
|  | National Institutes of Health Patient- Reported Outcomes Measurement Information System (NIH PROMIS)^(7)^ |  |  |
|  | Nottingham Health Profile (NHP)^(18, 20, 23)^ | Spanish, Turkish | Italy, Spain, Turkey |
|  | Observing QOL in Dementia (OQOLD) and Observing QOL for Dementia Advanced (OQOLDA)^(14)^ | English |  |
|  | Organ Transplant Symptom and Well-being Instrument (OTSWI)^(26)^ |  |  |
|  | Palliative Care Outcome Scale (POS)^(5-7, 15, 27)^ | English, German, Malayalam, Portugese, Spanish, Argentinean, Dutch, Chinese, French, Italian, Khmer, Punjabi, Urdu | USA UK, Germany, Africa, Denmark, France, Netherlands, Portugal, Spain, India, Singapore, Cambodia, Dominican Republic, Argentina, Cuba |
|  | Palliative Care Quality of Life Instrument (PQLI)^(15, 28)^ | Greek | Greece, Cyprus |
|  | Parkinson’s Disease Quality of Life (PDQ-39/PDQ-8)^(29)^ |  |  |
|  | Patient Autonomy Questionnaire (PAQ)^(5)^ |  |  |
|  | Patient-Evaluated Problem Scores (PEPS)^(7)^ |  |  |
|  | Progressive Deterioration Scale^(14)^ | English |  |
|  | Prostate Cancer Specific Quality of Life Instrument (PROSQOLI)^(22)^ | English | Canada |
|  | Psychological Well-being in Cognitively Impaired Persons (PWBCIP)^(14)^ | English | USA |
|  | QUALIDEM^(14, 17)^ | Dutch, German | Netherlands, Germany |
|  | Quality of Life and Health Questionnaire (QLHQ)^(7)^ |  |  |
|  | Quality of life assessment scale for gastric cancer patients (QLASTCM-Ga)^(9)^ |  | Mainland China |
|  | Quality of Life Assessment Schedule (QOLAS)^(14)^ | English | UK |
|  | Quality of life at the EOL (QUAL-E/QUAL-E Fam)^(5, 7, 19, 24, 28)^ | USA |  |
|  | Quality of Life in Alzheimer's Disease (QOL-AD)^(14, 17, 19, 29)^ | French, Portuguese, Spanish, Japanese, Cantonese, Mandarin, Korean | UK, Japan, Korea, Taiwan, Singapore, Australia, Spain, France, Switzerland, Brazil, Finland, Canada, Denmark, Australia, Norway, USA |
|  | Quality of Life in Late-Stage Dementia (QUALID)^(14, 17)^ | English, Swedish, Dutch, Spanish, Norwegian | Norway USA, Sweden, Netherlands, Spain |
|  | Quality of Life in Life-Threatening Illness - Patient version (QOLLTI-P)^(7)^ |  |  |
|  | Quality of Life Index (QL/QLI 3.0I)^(7, 18, 20, 22, 26)^ | Persian, English, Arabic, Turkish | Iran, USA, Turkey, Korea |
|  | Quality of Life Questionnaire for Dementia (QOL-D)^(14)^ | English | Japan, US |
|  | ReTransQoL (RTQ v1/RTQ v2)^(18)^ | French | France |
|  | Schedule for the Evaluation of Individual Quality of Life (SEIQoL)^(24)^ |  |  |
|  | Self-reported 11-point QOL scale^(19)^ |  |  |
|  | Sickness Impact Profile (SIP)^(20, 23, 26)^ |  | China |
|  | Spine Oncology Study Group Outcomes Questionnaire (SOSG-OQ)^(7)^ | English | USA, Netherlands |
|  | Spitzer Quality of Life Index (SQLI)^(22)^ |  |  |
|  | St. Christopher’s Index of Patient Priorities (SKIPP)^(6)^ |  | Mainland China, Hong Kong, Taiwan |
|  | Structured Interview for Symptoms and Concerns in PC (SISC)^(2)^ | English | Italy, USA |
|  | Transplant Care Index (TCI)^(26)^ |  |  |
|  | University of Washington Quality of Life Chinese Version (UWQOL-C)^(9)^ | Chinese | Taiwan |
|  | WHO Quality of Life (WHOQOL/WHOQOL-BREF)^(16, 18, 22-24, 26)^ | Tamil, Taiwanese | India, Taiwan, Sudan, Brazil |

**Table A6. Languages and Countries for measures assessing care experience, quality of dying and suffering**

| **#** | **Measure** | **Languages** | **Countries** |
| --- | --- | --- | --- |
| **Care Experience** | | | |
| 1 | Bereaved Family Survey (BFS)^(28)^ | English | USA, Puerto Rico |
| 2 | Canadian Health Care Evaluation Project (CANHELP)^(28)^ | English | Canada |
| 3 | Care Evaluation Scale (CES/CES-10)^(1, 24, 28)^ | English, Japanese, Korean | Japan, Korea |
| 4 | Care of the Dying Evaluation (CODE)^(1, 28)^ | English | UK |
| 5 | Caregiver Evaluation of the Quality of End-of-Life care (CEQUEL)^(1)^ | English | USA |
| 6 | Caregiver Voice Survey^(28)^ | English | Canada |
| 7 | Chinese Patient Satisfaction Questionnaire (ChPSQ-9)^(9)^ | Chinese | Hong Kong |
| 8 | Considerate^(28)^ | English | USA |
| 9 | Consumer Assessment of Healthcare Providers and Systems (CAHPS) Cancer Care /Hospice Survey^(28)^ | English | USA |
| 10 | Consumer Quality Index Palliative Care (CQ-Index-PC)^(1, 28)^ | Dutch | Netherlands |
| 11 | Dementia Care Mapping (DCM)^(14)^ | English | UK, US |
| 12 | Dying Care Process Scale ^(28)^ |  | Japan |
| 13 | End-of-life in Dementia Scales - SWC-EOLD^(1, 28, 30)^ | Dutch, English | USA, Netherlands |
| 14 | EORTC Satisfaction with In-Patient Cancer Care (EORTC IN-PATSAT32)^(9)^ |  | Mainland China |
| 15 | euroQ2 Satisfaction with Care in the ICU^(28)^ |  | Denmark |
| 16 | Evaluating Care and Health Outcomes-for the Dying (ECHO-D)^(1)^ | English | UK |
| 17 | Family Assessment of Treatment of End-of-Life (FATE/FATE-S-14/FATE-S-12/FATE-32)^(1, 28, 30)^ | English, Spanish, Dutch | USA, Netherlands |
| 18 | Family Evaluation of Hospice Care (FEHC)^(1, 28)^ | English | USA |
| 19 | Family Evaluation of Palliative Care (FEPC)^(28)^ | English | USA |
| 20 | Family Perceptions of Care Scale (FPCS)^(1)^ | English, Dutch | USA, Canada, Netherlands |
| 21 | Family Perceptions of Physician-Family Caregiver Communication (FPPFC) ^(1)^ | English, Dutch | USA, Netherlands |
| 22 | Family Satisfaction in the ICU (FS-ICU)^(24, 28, 30, 31)^ | English | USA, Canada |
| 23 | Family Satisfaction with Advanced Cancer Care (FAMCARE) ^(1, 24, 28, 30, 32)^ | English, Portuguese | Norway, USA, Australia, Portugal |
| 24 | Family Satisfaction with Care Questionnaire^(31)^ | English | USA |
| 25 | Feeling Heard and Understood^(28)^ | English | USA |
| 26 | Health Competence Beliefs Inventory (HCBI)^(8)^ | English | USA |
| 27 | Maastricht Instrument on Satisfaction with Terminal Care (MITTZ)^(24)^ |  |  |
| 28 | McCusker EOLC scale^(1)^ | English | USA |
| 29 | Otani et al. 2020 study developed questionnaire^(28)^ |  | Japan |
| 30 | Patient and Family Satisfaction Survey^(31)^ | English | USA |
| 31 | Patient Satisfaction Questionnaire (PSQ)^(28)^ | English | UK |
| 32 | Postmortem Questionnaire-Short Form (QPM-SF)^(1)^ | Italian | Italy |
| 33 | Quality Care Questionnaire-Palliative Care (QCQ-PC)^(9, 28)^ |  | Korea, Mainland China |
| 34 | Quality from the Patient’s Perspective (QPP-PC)^(28)^ |  | Norway |
| 35 | Quality Measure for Palliative Nursing^(6)^ | English | UK |
| 36 | Quality of Communication Questionnaire (QOC)^(6, 28)^ | English | USA |
| 37 | Quality of End-of-Life Care and Satisfaction with Treatment (QUEST)^(24, 28, 30)^ | English | USA |
| 38 | Quality of End-of-Life Care Questionnaire (QEOLC)^(6, 28)^ | English | USA |
| 39 | Quality of oncology nursing care scale (QONCS)^(9)^ |  | Mainland China |
| 40 | Reid-Gundlach Satisfaction with Services instrument^(31)^ | English | USA |
| 41 | Satisfaction Scale for Family members receiving Inpatient Palliative Care (Sat-Fam-IPC)^(1, 28, 30)^ | English | Japan |
| 42 | Satisfaction with care Scale^(24)^ |  |  |
| 43 | Satisfaction with Doctors Questionnaire^(28)^ |  | Turkey |
| 44 | Sense of Security in Care – Patients’ Evaluation (SEC-P)^(6)^ |  | Sweden |
| 45 | The Sinclair Compassion Questionnaire (SCQ)^(28)^ | English | Canada |
| 46 | Toolkit After-Death Bereaved Family Member Interview^(1, 28, 30)^ | English, Dutch | USA, Netherlands |
| 47 | Users’ Satisfaction with Nursing Care instrument (SUCEH21)^(32)^ | Portuguese | Portugal |
| 48 | Victorian Palliative Care Satisfaction Instrument (VPCSI)^(28)^ | English | Australia |
| 49 | Views of Informal Carers Evaluation of Services (VOICES)^(28, 30)^ | English | UK |
| **Quality of Dying** | | | |
| 1 | Cohen et al 2005^(21)^ | English | USA |
| 2 | Dialysis Discontinuation Quality of Dying (DDQOD)^(21, 33)^ | English | USA, Canada |
| 3 | Dying Care Outcdome Scale ^(28)^ |  | Japan |
| 4 | End-of-life in Dementia Scales - CAD-EOLD^(1, 24, 28, 30)^ | Dutch, English | USA, Netherlands |
| 5 | Flacker et al 2001^(21)^ | English | USA |
| 6 | Ganzini et al 2003^(21)^ |  | USA |
| 7 | Good Death Inventory (GDI/GDI-sv)^(1, 12, 21, 33)^ | Japanese, Korean, Chinese | Japan, Iran, Korea, China, Taiwan |
| 8 | Good Death Scale (GDS)^(12, 21, 33)^ | Taiwanese, Chinese | Taiwan |
| 9 | McCanse Readiness for Death Instrument (MRDI)^(5)^ |  |  |
| 10 | Peruselli et al 1999^(21)^ |  | Italy |
| 11 | Quality of Dying (QOD-Hospice/ QOD-LTC)^(1, 21, 33)^ | English, German, Spanish | USA, Netherlands |
| 12 | QODD Questionnaire (QODD/QODD-ESP/QODD-D-ANG/QODD-Chinese/QODD-Korean)^(1, 5, 12, 21, 24, 28, 30, 31, 33)^ | Spanish, English, Chinese, German, Korean | Chile, USA, China, Germany, Korea |
| 13 | Ray et al. 2006^(21)^ | English | USA |
| 14 | Reynolds et al 2002^(21)^ |  | USA |
| 15 | The Quality of Death^(21)^ | English | USA |
| **Suffering** | | | |
| 1 | Cancer Distress Scales for Adolescents and Young Adults (CDS-AYA)^(8)^ | English | Canada |
| 2 | Initial Assessment of Suffering (IAS)^(2, 11)^ | English | Australia |
| 3 | Measurement Instrument for Dignity Amsterdam (MIDAM)^(34)^ | Dutch | Netherlands |
| 4 | Mini-Suffering State Examination (MSSE)^(2, 11)^ | English | Israel |
| 5 | Palliative Patient's Dignity Scale (PPDS)^(34)^ | Spanish | Spain |
| 6 | Patient Dignity Inventory (PDI)^(5, 7, 34)^ | English, Turkish, Brazillian, Mexican, Swedish, Tamil, Italian, Czech, Mandarin, Spanish, Persian, German, Dutch, Greek | USA, Turkey, Brazil, Mexico, Sweden, India, Italy, Taiwan, Czech Republic, Greece, China, Spain, Iran, Canada, Netherlands, Germany |
| 7 | Peace, Equanimity, and Acceptance in the Cancer Experience (PEACE)^(7)^ |  |  |
| 8 | Pictorial Representation of Illness and Self-Measure (PRISM/PRISM-R1/PRISM-R2)^(2, 11)^ | English, German, Spanish | Netherlands, Colombia |
| 9 | State Of Suffering-Five (SOS-V)^(2, 11)^ | English |  |
| 10 | Suffering Assessment Questionnaire in Adults with Chronic Diseases or Life-Threatening Illness (SAQ)^(11)^ | English | UK |
| 11 | Suffering Assessment Tool (SAT)^(2)^ | English |  |
| 12 | Suffering Scale^(2)^ | English |  |
| 13 | The Suffering Pictogram^(11)^ | English | Malaysia |
| 14 | The Suffering Scales^(2)^ | English |  |

**Table A7. Methodological qualities of measures with sufficient psychometric evidence**

| **#** | **Measure** | **Disease group** | **Internal consistency** | **Reliability** | **Content validity** | **Structural validity** | **Hypothesis testing** | **Responsiveness** | | **Cross cultural validity** | | **Criterion validity** | **Measurement error** |
| --- | --- | --- | --- | --- | --- | --- | --- | --- | --- | --- | --- | --- | --- |
| ***Quality of Life*** | | | | | | | | | | | | | |
|  | Cancer Module Adolescent Form^ | Cancer, blood disorders | Yes^(8)^ |  |  |  | Yes^(8)^ |  | |  | |  |  |
|  | Generic Core Scales Adolescent Form^ | Cancer, blood disorders | Yes^(8)^ |  |  |  | Yes^(8)^ |  | |  | |  |  |
| ***Care experience*** | | | | | | | | | | | | | |
|  | Care Evaluation Scale (CES/CES-10) | Cancer |  | Yes^(1)^ | Yes^(1)^ |  |  |  | |  | |  |  |
|  | Caring Of the Dying Evaluation (CODE) | Generic | Yes^(1)^ |  | Yes^(1)^ |  |  |  | |  | |  |  |
|  | Consumer Quality Index Palliative Care (CQ-Index-PC) | Generic |  |  | Yes^(1)^ |  |  |  | |  | |  |  |
|  | Evaluating Care and Health Outcomes-for the Dying (ECHO-D) | Cancer | No^(1)^ |  |  |  | No^(1)^ |  | |  | |  |  |
|  | End-of-life in Dementia Scales – Satisfaction with Care (SWC-EOLD) | Dementia | Yes^(1)^ |  |  |  |  |  | |  | |  |  |
|  | FATE-32 | Generic | No^(1)^ |  | Yes^(1)^ |  |  |  | |  | |  |  |
|  | Family Perceptions of Care Scale (FPCS) | Generic |  |  | No^(1)^ | No^(1)^ |  |  | |  | |  |  |
|  | Family satisfaction with end-of-life care (FAMCARE) | Cancer | No^(1)^ |  |  | No^(1)^ |  |  | |  | |  |  |
|  | Health Competence Beliefs Inventory (HCBI)^ | Cancer | Yes^(8)^ |  |  |  | No^(8)^ |  | |  | |  |  |
|  | Postmortem Questionnaire-Short Form (QPM-SF) | Cancer |  |  | Yes^(1)^ | No^(1)^ |  |  | |  | |  |  |
|  | Satisfaction Scale for Family members receiving Inpatient Palliative Care (SAT-Fam-IPC) | Generic |  |  | Yes^(1)^ | Yes^(1)^ |  |  | |  | |  |  |
| ***Quality of dying*** | | | | | | | | | | | | | |
|  | QODD- Korean | Generic | Yes^(12)^ |  |  |  |  |  |  | |  | |  |
|  | QODD -Chinese | Generic | Yes^(12)^ |  |  | Yes^(12)^ |  |  |  | |  | |  |
| ***Suffering*** | | | | | | | | | | | | | |
|  | Cancer Distress Scales for Adolescents and Young Adult^ | Cancer |  | No^(8)^ | No^(8)^ |  | No^(8)^ | Yes^(8)^ | Yes^(8)^ | |  | |  |
|  | Mini-Suffering State Examination (MSSE) | Generic |  | No^(11)^ |  |  |  |  |  | | No^(11)^ | |  |
|  | The Suffering Pictogram | Generic | Yes^(11)^ |  | Yes^(11)^ |  |  |  |  | |  | |  |

Yes (low risk) or No (high risk), None of the measures with sufficient psychometric evidence reported having inconsistent methodological quality.

^ Young adults

**References**

1. Kupeli N, Candy B, Tamura-Rose G, Schofield G, Webber N, Hicks SE, et al. Tools Measuring Quality of Death, Dying, and Care, Completed after Death: Systematic Review of Psychometric Properties. Patient. 2019;12(2):183-97.

2. Krikorian A, Limonero JT, Corey MT. Suffering assessment: a review of available instruments for use in palliative care. J Palliat Med. 2013;16(2):130-42.

3. Bot SD. Psychometric evaluation of self-report questionnaires: the development of a checklist. Proceedings of the second workshop on research methodology. 2003:161-8.

4. Terwee CB, Bot SD, de Boer MR, van der Windt DA, Knol DL, Dekker J, et al. Quality criteria were proposed for measurement properties of health status questionnaires. J Clin Epidemiol. 2007;60(1):34-42.

5. Albers G, Echteld MA, de Vet HC, Onwuteaka-Philipsen BD, van der Linden MH, Deliens L. Evaluation of quality-of-life measures for use in palliative care: a systematic review. Palliat Med. 2010;24(1):17-37.

6. Kearns T, Cornally N, Molloy W. Patient reported outcome measures of quality of end-of-life care: A systematic review. Maturitas. 2017;96:16-25.

7. van Roij J, Fransen H, van de Poll-Franse L, Zijlstra M, Raijmakers N. Measuring health-related quality of life in patients with advanced cancer: a systematic review of self-administered measurement instruments. Qual Life Res. 2018;27(8):1937-55.

8. Chambers RL, Hocaoglu MB, Higginson IJ, Sleeman KE, Fraser LK. Evaluating the measurement properties of patient-reported outcome measures for young adults with life-limiting conditions: A systematic review. Palliat Med. 2025;39(7):765-91.

9. Li H, Guo P, Gao W, Normand C, Harding R. Patient-reported outcome measures for advanced cancer in China: A systematic review of cross-cultural adaptation and psychometric properties. J Cancer Policy. 2023;35:100371.

10. Prinsen CAC, Mokkink LB, Bouter LM, Alonso J, Patrick DL, de Vet HCW, et al. COSMIN guideline for systematic reviews of patient-reported outcome measures. Qual Life Res. 2018;27(5):1147-57.

11. Gutierrez-Sanchez D, Gomez-Garcia R, Cuesta-Vargas AI, Perez-Cruzado D. The suffering measurement instruments in palliative care: A systematic review of psychometric properties. Int J Nurs Stud. 2020;110:103704.

12. Xu S, Fang Y, Chen H, Sun K, Zhang C, Liu Y. A systematic review of instruments measuring the quality of dying and death in Asian countries. Qual Life Res. 2023;32(7):1831-42.

13. Terwee CB, Prinsen CAC, Chiarotto A, Westerman MJ, Patrick DL, Alonso J, et al. COSMIN methodology for evaluating the content validity of patient-reported outcome measures: a Delphi study. Qual Life Res. 2018;27(5):1159-70.

14. Bowling A, Rowe G, Adams S, Sands P, Samsi K, Crane M, et al. Quality of life in dementia: a systematically conducted narrative review of dementia-specific measurement scales. Aging Ment Health. 2015;19(1):13-31.

15. Correia FR, De Carlo MM. Evaluation of quality of life in a palliative care context: an integrative literature review. Rev Lat Am Enfermagem. 2012;20(2):401-10.

16. Potts M, Cartmell KB, Nemeth L, Bhattacharjee G, Qanungo S. A Systematic Review of Palliative Care Intervention Outcomes and Outcome Measures in Low-Resource Countries. J Pain Symptom Manage. 2018;55(5):1382-97 e7.

17. Burks HB, des Bordes JKA, Chadha R, Holmes HM, Rianon NJ. Quality of Life Assessment in Older Adults with Dementia: A Systematic Review. Dement Geriatr Cogn Disord. 2021;50(2):103-10.

18. Aiyegbusi OL, Kyte D, Cockwell P, Marshall T, Gheorghe A, Keeley T, et al. Measurement properties of patient-reported outcome measures (PROMs) used in adult patients with chronic kidney disease: A systematic review. PLoS One. 2017;12(6):e0179733.

19. Li Y, Hung V, Ho K, Kavalieratos D, Warda N, Zimmermann C, et al. The Validity of Patient-Reported Outcome Measures of Quality of Life in Palliative Care: A Systematic Review. J Palliat Med. 2024;27(4):545-62.

20. Peng JK, Hepgul N, Higginson IJ, Gao W. Symptom prevalence and quality of life of patients with end-stage liver disease: A systematic review and meta-analysis. Palliat Med. 2019;33(1):24-36.

21. Hales S, Zimmermann C, Rodin G. Review: the quality of dying and death: a systematic review of measures. Palliat Med. 2010;24(2):127-44.

22. Roydhouse JK, Wilson IB. Systematic review of caregiver responses for patient health-related quality of life in adult cancer care. Qual Life Res. 2017;26(8):1925-54.

23. Glover C, Banks P, Carson A, Martin CR, Duffy T. Understanding and assessing the impact of end-stage renal disease on quality of life: a systematic review of the content validity of self-administered instruments used to assess health-related quality of life in end-stage renal disease. Patient. 2011;4(1):19-30.

24. Stiel S, Pastrana T, Balzer C, Elsner F, Ostgathe C, Radbruch L. Outcome assessment instruments in palliative and hospice care--a review of the literature. Support Care Cancer. 2012;20(11):2879-93.

25. Hounsome N, Orrell M, Edwards RT. EQ-5D as a quality of life measure in people with dementia and their carers: evidence and key issues. Value Health. 2011;14(2):390-9.

26. Mahmoudi R, Moitie T, Dorent R, Guillemin F, Couchoud C. Implementation of patient-reported outcome measures in a heart transplant recipient registry: First step toward a patient-centered approach. Clin Transplant. 2022;36(8):e14708.

27. Bausewein C, Le Grice C, Simon S, Higginson I, Prisma. The use of two common palliative outcome measures in clinical care and research: a systematic review of POS and STAS. Palliat Med. 2011;25(4):304-13.

28. Virdun C, Garcia M, Phillips JL, Luckett T. Description of patient reported experience measures (PREMs) for hospitalised patients with palliative care needs and their families, and how these map to noted areas of importance for quality care: A systematic review. Palliat Med. 2023;37(7):898-914.

29. Holden SK, Jones WE, Baker KA, Boersma IM, Kluger BM. Outcome measures for Parkinson's disease dementia: a systematic review. Mov Disord Clin Pract. 2016;3(1):9-18.

30. Lendon JP, Ahluwalia SC, Walling AM, Lorenz KA, Oluwatola OA, Anhang Price R, et al. Measuring Experience With End-of-Life Care: A Systematic Literature Review. J Pain Symptom Manage. 2015;49(5):904-15 e1-3.

31. Quigley DD, McCleskey SG. Improving Care Experiences for Patients and Caregivers at End of Life: A Systematic Review. Am J Hosp Palliat Care. 2021;38(1):84-93.

32. Ferreira A, Pereira A, Pinto S. Satisfaction with Care Received at the End of Life in Portugal: A Systematic Review. Nurs Rep. 2025;15(6).

33. Gutierrez Sanchez D, Perez Cruzado D, Cuesta-Vargas AI. The quality of dying and death measurement instruments: A systematic psychometric review. J Adv Nurs. 2018.

34. Lam LT, Chang HY, Natashia D, Lai WS, Yen M. Self-report instruments for measuring patient dignity: A psychometric systematic review. J Adv Nurs. 2022;78(12):3952-73.
